# Supplementary material for: Inherent biomechanical traits enable infective filariae to disseminate through collecting lymphatic vessels
Source: Nat Commun. 2019 Jul 1;10:2895. doi: 10.1038/s41467-019-10675-2 (PMC6603047; doi:10.1038/s41467-019-10675-2)
Supplement: Supplementary file 1 — Supplementary Information [file 41467_2019_10675_MOESM1_ESM.pdf]

Supplementary information

Inherent biomechanical traits enable infective filariae to disseminate through collecting lymphatic vessels

Witold W. Kilarski[1,2,*,Ω], Coralie Martin[3],Marco Pisano[2], Odile Bain[3†], Simon Babayan[4], and Melody A. Swartz[1, 2, 5,*,Ω]

Supplementary Figures

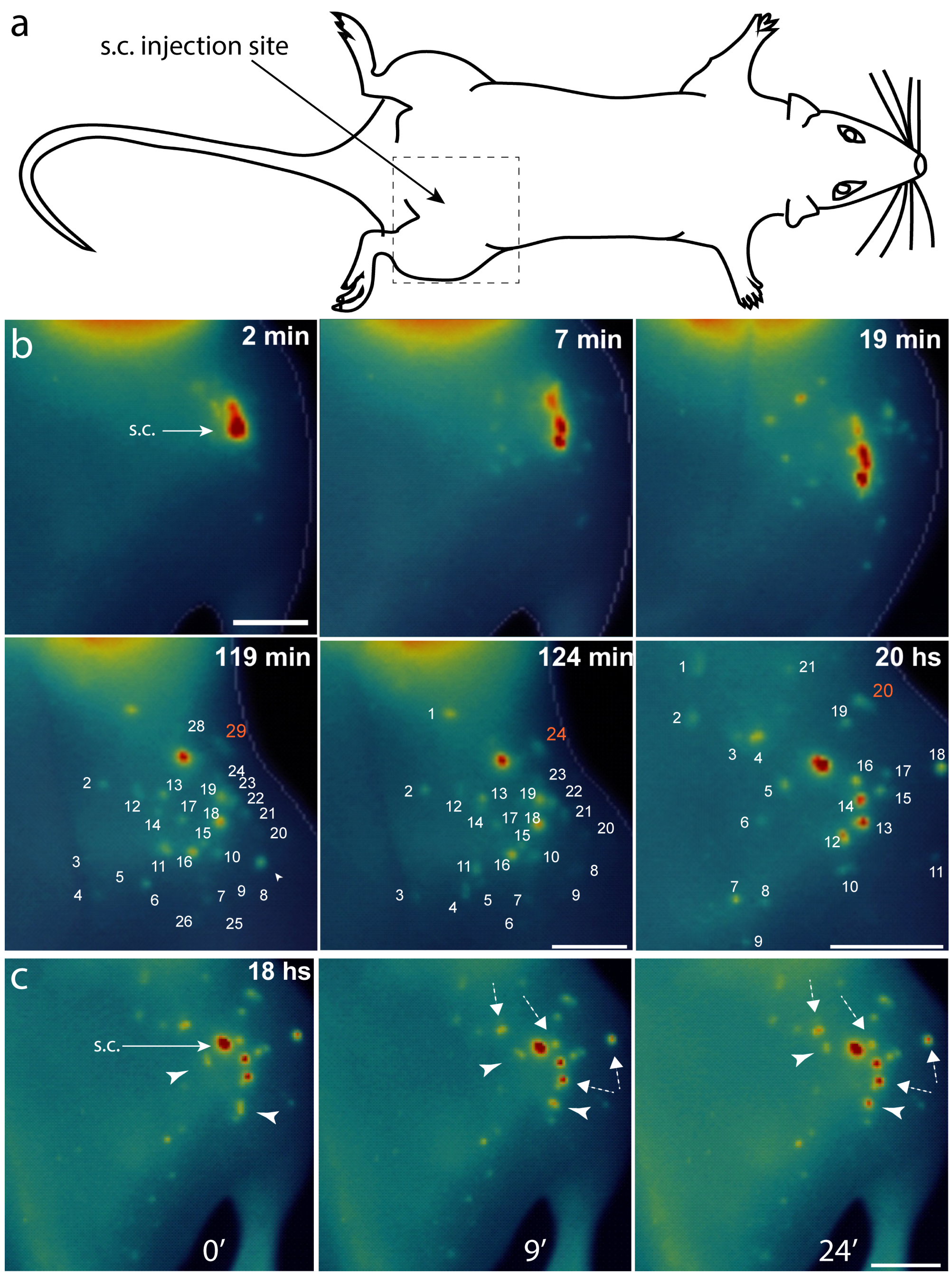

Supplementary Figure 1. Labeled filariae escape the subdermal inoculation site

a. Schematic showing the location within the mouse imaged in (b) and (c) panels. A dashed box denotes the imaging area. **b-c.** Macroscopic timelapse imaging of near-infrared-tag-labeled L3 larvae inoculated at the mouse lumbar area under the dorsolateral skin. (b) and (c) show experiments performed on different mice. **b.** Analysis of the spread of labeled filariae from subcutaneous inoculation site (Supplementary Movie 1). Out of 50 filariae loaded into the syringe, 39 were successfully deposited under the skin. The subcutaneous spread of larvae reached its maximum within 2 h after the inoculation as compared with their distribution 18 h later. 9 larvae left the dermis between 2 and 20 h after the inoculation. Between 119 and 124 min, bright filaria (arrowhead pointing to filaria 9) escaped from the imaging field. Numbers denote the manually counted filaria larvae spreading under the dermis. Numbers in red show the highest larvae count at the given time. Between 19 and 119 min, and 119 min and 20 h mouse was returned to the cage hence its shape during subsequent imaging changed. **c.** Time-lapse imaging of filariae 18 h after subcutaneous inoculation. Arrowheads point to 2 migrating filariae; dashed arrows point to 4 wiggling filariae (as inferred from changes in their fluorescent intensity). The lookup table represents a single imaging near-infra-red channel with the blue color denoting a minimal signal from mouse autofluorescence and the red, strong signal from filaria and autofluorescence of the liver. Scale bars: 5 mm.

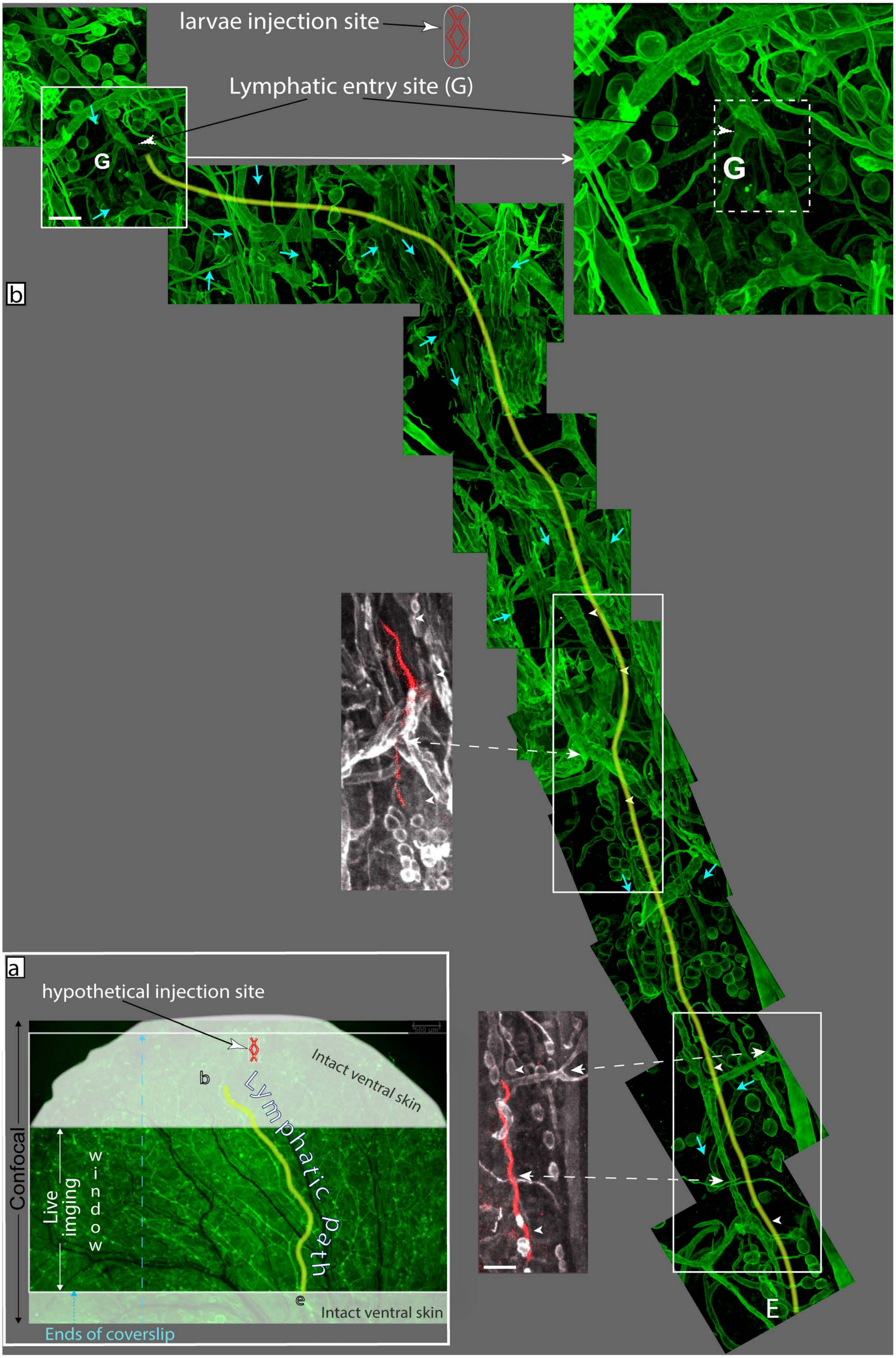

larvae injection site
Lymphatic entry site (G)
G
b
E
a
hypothetical injection site
Intact ventral skin
Lymphatic path
Confocal
Live imaging window
Intact ventral skin
Ends of coverslip

Supplementary Figure 2. Complete intra-lymphatic migratory path of the filaria (movie 9)

a. Schematic of the ear imaging window and the example marking of the complete path of the lymphatic pre-collectors. A model image of the dorsal dermis of a mouse ear live-stained for collagen IV (Coll IV) was taken from the Supplementary Fig. 8. A yellow line is drawn right to the lymphatic collector that ran through the dorsal dermis from the beginning of pre-collector (b) to its end (e). The distal and proximal fragments (white-painted dorsal dermis) delineate the ear imaging window. The distal (top) fragment of the intact ventral skin protected the dorsal dermis during the inoculation.**b.** High-resolution reconstruction of the L3 filaria intralymphatic migratory path from Supplementary movie 9. Before the inoculation of filariae, a fragment of ventral skin with underlying cartilage from the middle part of the ear was removed, and the exposed dorsal dermis was live-stained for collagen IV. After that, filaria larvae were injected in the top, distal to the head, fragment of the dorsal skin that was covered with the intact ventral dermis and the cartilage. Within 3 min after the inoculation, time-lapse imaging of the exposed dorsal dermis started in 647nm (collagen IV) and red (546-594nm) channels. 12 h later the mouse was fix-perfused, and re-stained skin was imaged in a confocal microscope. The confocal maximum intensity projection images were used to reconstruct the migratory path of the filaria. Migratory path is marked in yellow as described in panel (a). Except for the G-site, no other injuries were found in connected lymphatics. The confocal reconstruction (green, fixed tissue) shows only collagen IV staining. Two gray images of collagen IV and red-migrating filaria were taken with epifluorescence stereomicroscope during intravital imaging and serve as reference points for the confocal reconstruction. Arrowheads and double-sided dashed arrows point to the same reference locations. Blue arrow point to the predicted direction of lymph flow within relevant lymphatic pre-collectors. Scale bars: 100 µm.

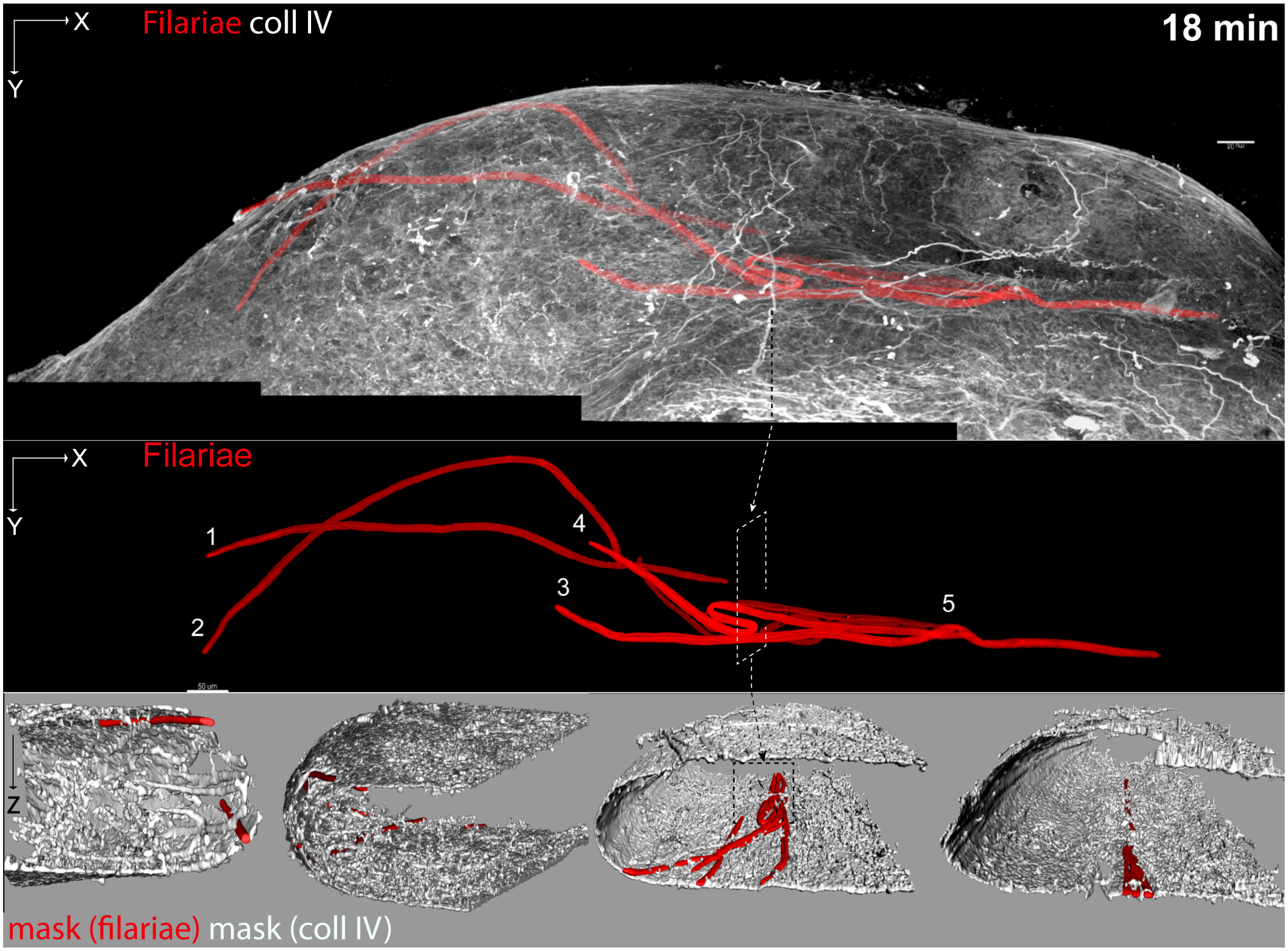

Supplementary Figure 3. Lymph node subcapsular sinus trapped migrating filariae
Five filaria larvae in the subcapsular sinus of the first (superficial cervical) lymph node (LN) draining the ear. Infectious L3 larvae reached the draining LN 18 min after the inoculation. **Top**. Maximum intensity projection image of the LN stained for collagen IV (Coll IV). **Middle**. The same image as in Top without collagen IV channel showing morphological details of 5 larvae. **Bottom**. Surface masking over LN collagen IV and filariae channels revealed the subcapsular localization of all 5 larvae. Scale bars: 50 μm.

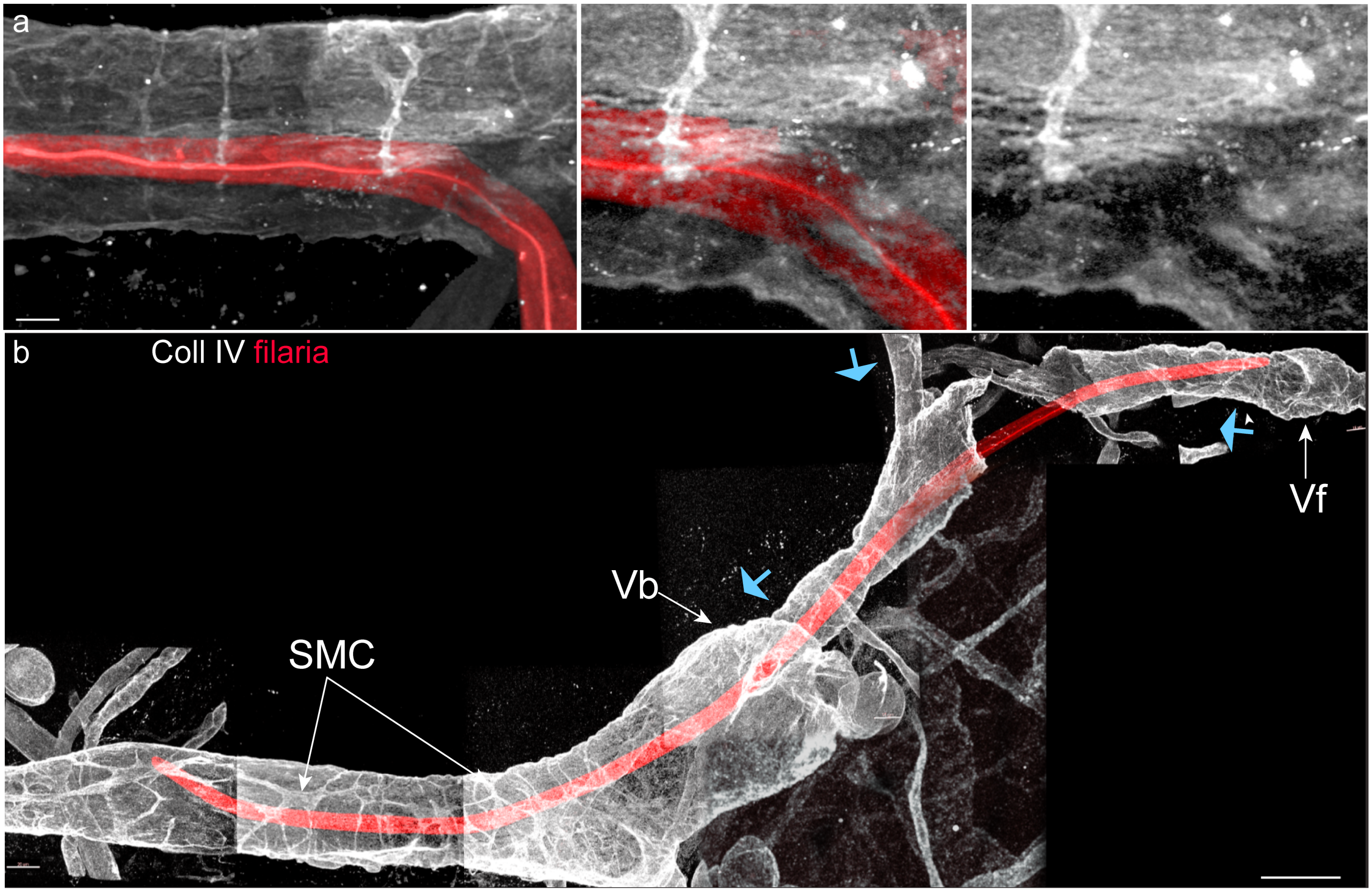

Supplementary Figure 4. Filariae break on through the wall of lymphatic collectors

a. The puncture wound marked in the lymphatic basement membrane with a larva captured partially within lymphatic pre-collector during the entry process. **b.** The filaria that entered the lymphatic collector was oriented in the direction opposing the valve system. Note the obliterating damage to the lymphatic pre-collector that separates the vessel near the head of the larva. Vb – bicuspid valve, Vf – funnel valve, SMC-smooth muscle cells imprints over the pre-collector basement membrane. Blue arrows point to the inferred direction of lymph flow. Scale bars: a, 10 µm, b, 100 µm.

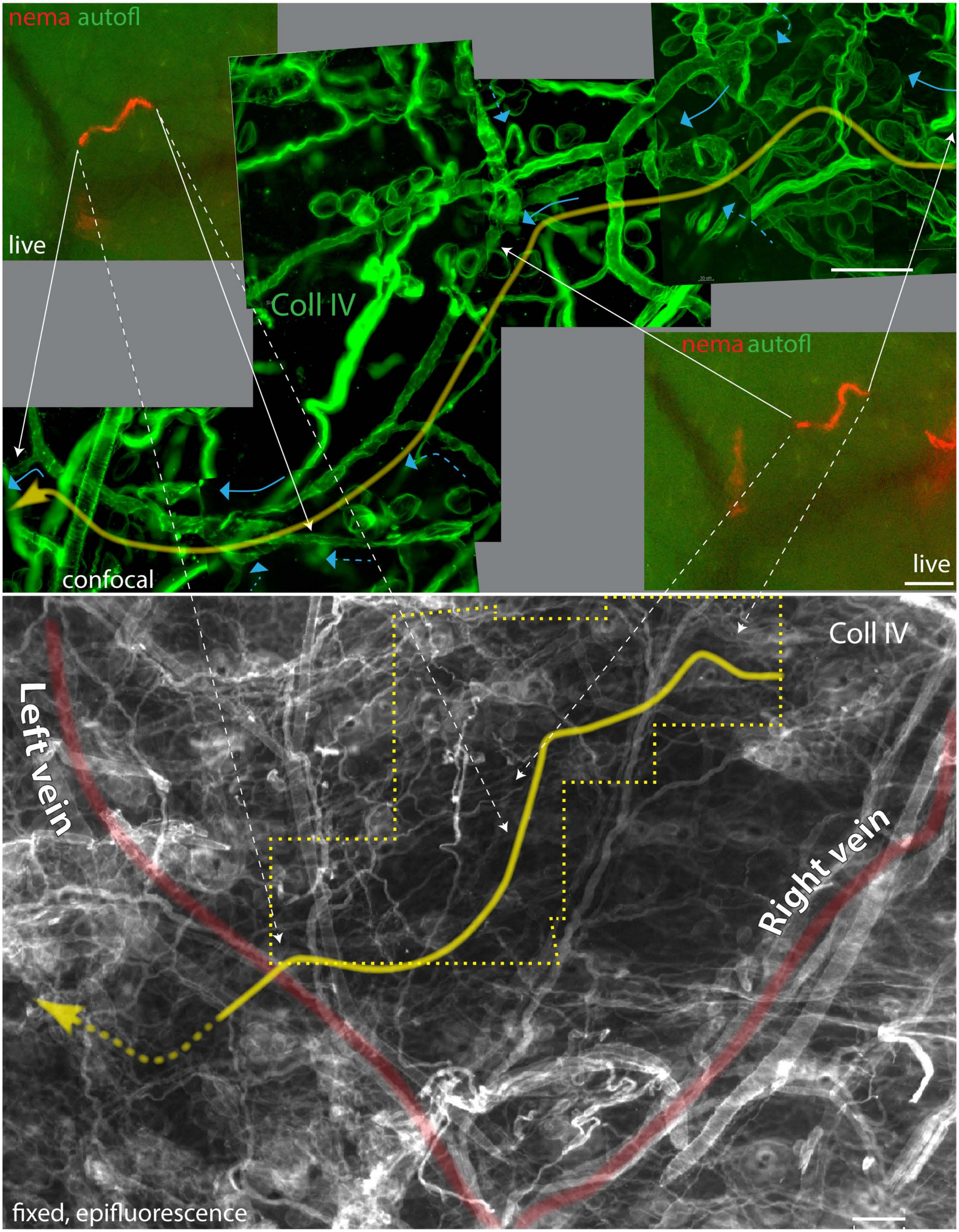

nema autofl
live
Coll IV
nema autofl
live
confocal
Coll IV
Left vein
Right vein
fixed, epifluorescence

Supplementary Figure 5. Migratory path of the nematode from movies 18 and 19

The dorsal ear skin was injected intradermally with the fluorescently labeled soil-derived nematode. Because of its rapid and unidirectional migration, the nematode that left the inoculation pocket as a second in Supplementary movie 18 was traced in Fig. 5a. 5 h after acquiring that intravital imaging sequence the mouse was fixed, the dorsal dermis was stained for collagen IV (Coll IV) and imaged in epifluorescence and confocal microscopes. **Top** (Coll IV, green). Reconstruction of the migratory path of the nematode from confocal maximum intensity projection images of collagen IV staining. The predicted lymph from inferred from the organization of the valve is indicated with the blue arrows. Two referenced (live) images (nema, nematode, autofl, tissue autofluorescence) are from the intravital imaging sequence done prior to tissue fixation and staining. **Bottom** (Coll IV, grey). Overview epifluorescence stereomicroscope reference image of the same dermis as in the (Top) reconstruction taken after the tissue was fixed and stained for collagen IV. The yellow line runs below (or right to) the lymphatic pre-collector path that was likely used by the nematode (the marking method described in Supplementary Fig. 2a). The dotted yellow arrow represents a possible collector route that could not be imaged due to tissue damage that occurred post-fixation. The dotted shape represents the area in the confocal reconstruction shown in the (Top) panel. Scale bars: 100 μm.

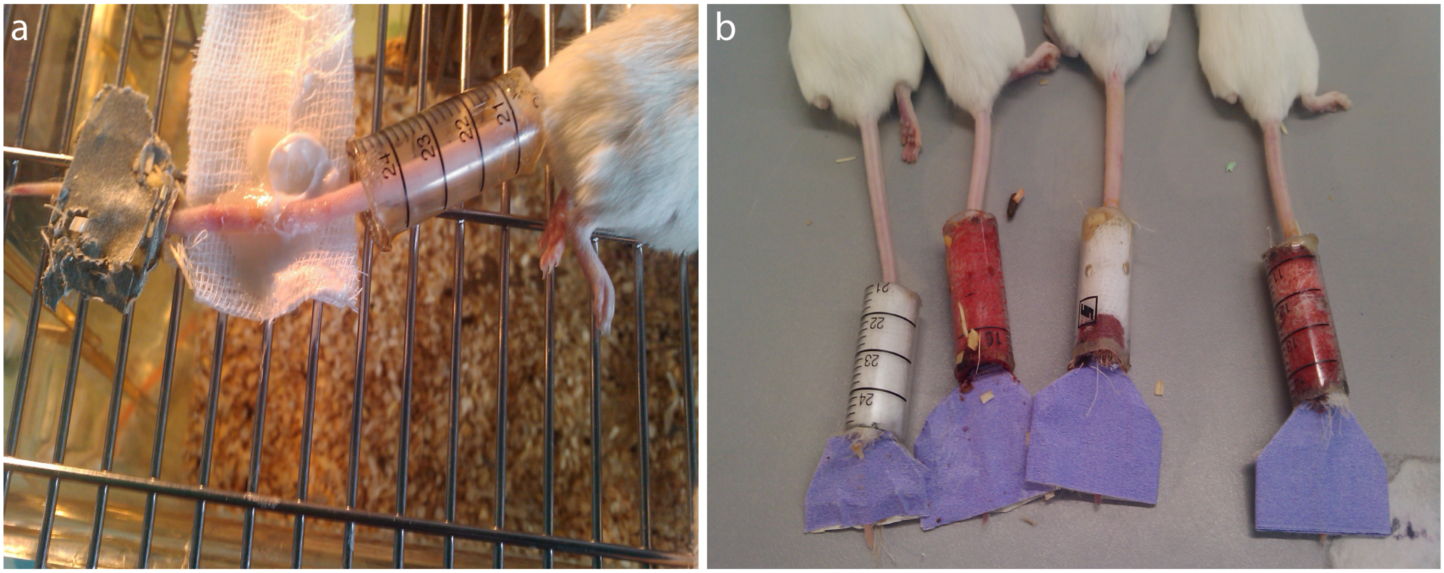

Supplementary Figure 6. Aseptic dressing of tail wound (secondary lymphedema)
The wound isolation and protection is a necessary measure to avoid infection of the wound that is in constant contact with cage bedding. **a.** Mouse tail during the dressing change 3 days after the surgery. To inspect the wound and change the dressing, the plastic tube that protected the wound from self-grooming, infections, and injuries was slid towards the base of the tail. The wound was directly protected by the tight silicon sleeve, and moisture was sustained with a bandage soaked in Bethaphen cream. At the end of the tail, multiple layers of paper tape (here partially chewed) kept the plastic tubing in place over the wound during regular mouse activity. **b.** Hemorrhaging from two tail wounds 3 days after the surgery. Without cauterization, non-hemorrhaging wounds started bleeding when the physiological blood pressure was restored in awake animals. The bleeding intensity decrease in time but wounds might bleed for 2 to 5 days after the surgery.

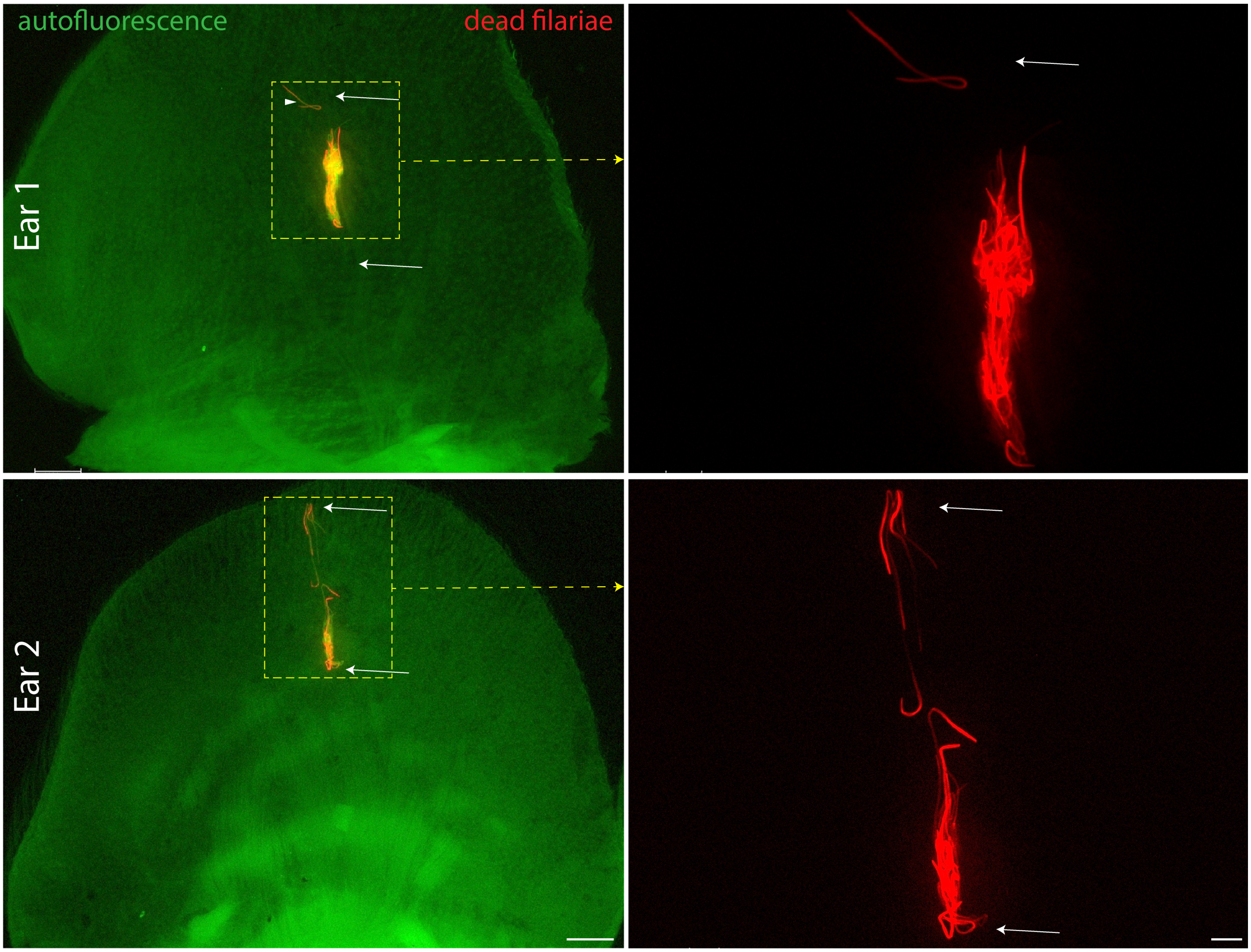

Supplementary Figure 7. Intradermal injection could not force filariae into the interstitium

To verify that filariae entry into the dermis is not forced by filaria-independent passive mechanical forces induced by the transient injection-driven edema, 50 dead (freeze-thawed) TRITC-labelled filariae into the dorsal ear skin. After 3 h mice were fixed-perfused, and skin flaps were separated and imaged. The larvae were all confined to the needle inoculation pocket (arrows), which collapsed after the dorsal and cartilage-supported-ventral skin flaps were separated. Arrowhead indicates a larva that was moved outside the inoculation pocket during the separation of skin flaps. Scale bars: Left, 1 mm; Right, 250 µm.

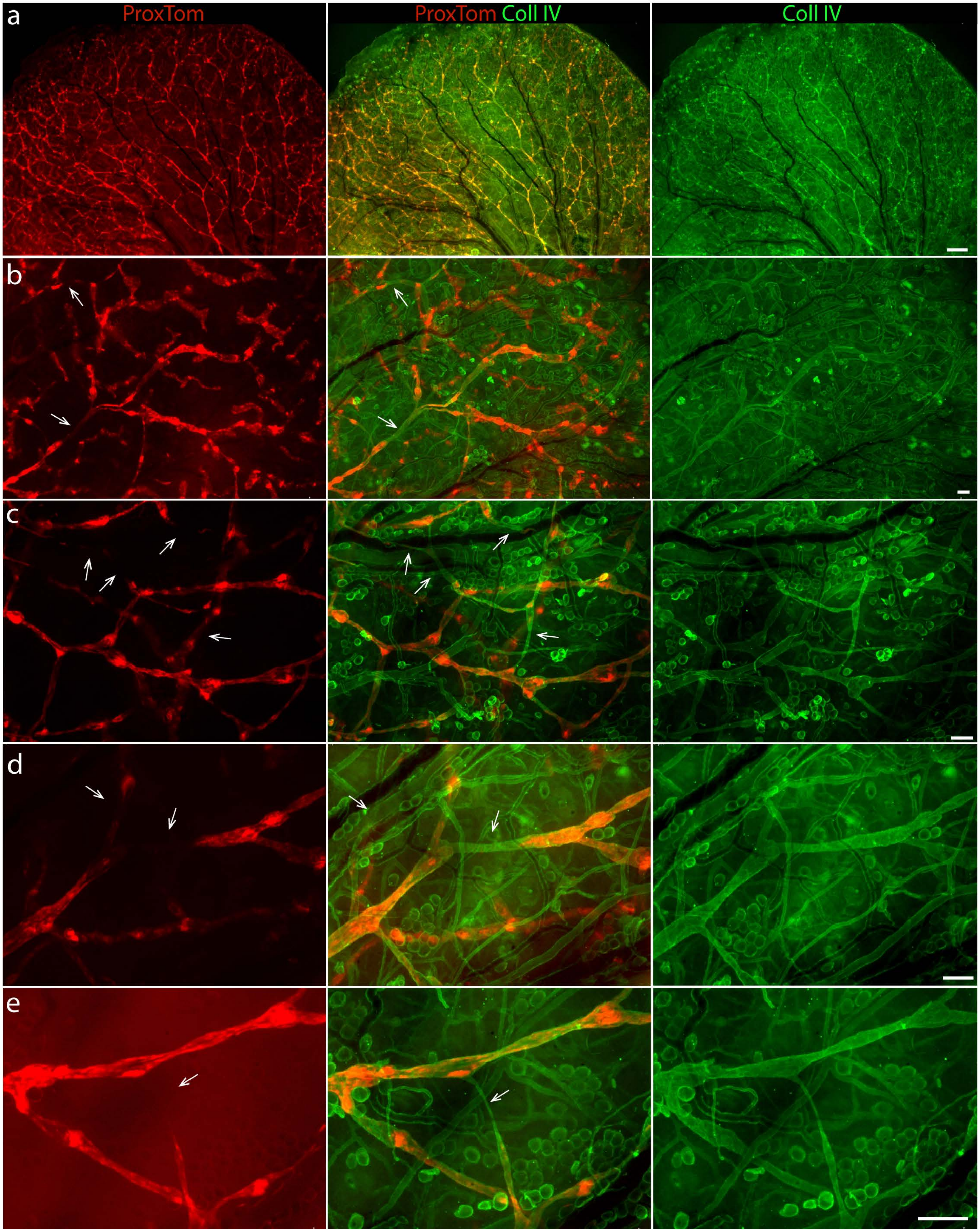

a
ProxTom
ProxTom Coll IV
Coll IV
b
c
d
e

Supplementary Figure 8. Basement membrane is a reliable marker of collector continuity

Intravital imaging of the lymphatics in the ear dorsal dermis in Prox1-Cre-tdTomato (ProxTom) reporter mouse live-stained for collagen IV (Coll IV). Lymphatic endothelium expressed tdTomato protein in the Prox1-dependent fashion. The exposed ear was live-stained for collagen IV to reveal the basement membrane of the dermis. **a.** Low magnification shows the entire ear with ProxTom-labeled lymphatic networks and dark blood-filled veins and arteries. In the collagen IV-stained panels, blood-filled vasculature was readily distinguished from the network of lymphatic pre-collecting vessels. **b-e.** Higher magnification images from panel A show discontinuous expression of tdTomato protein within the same lymphatic pre-collecting vessels (arrows). Collagen IV staining indicated that tdTomato-negative lymphatic fragments were continuously joined with tdTomato-positive segments. The expression of tdTomato varied from weak but detectable **(b-c)** to completely absent **(d-e)**, while collagen IV staining was homogeneous along vessels and did not reflect the heterogeneous expression of the tdTomato protein. In **(e)**, the gamma of the red fluorescence channel was decreased to 0.5 (an exaggerated increase of midtones and shadows), which highlighted the absence of detectable expression of the tdTomato protein in the lymphatic pre-collector, otherwise evenly stained by collagen IV (arrow). Collagen IV outlines the external wall of the collector; therefore the heterogeneity of endothelial expression of tdTomato protein cannot be explained by the artifacts related to the ear preparation. The ear was live-stained and imaged, which also eliminated the risk of denaturing or proteolysis of the tdTomato protein that could dim the fluorescence during tissue fixation and clearing. Scale bars: a, 500 µm; b-e, 100 µm.

Supplementary methods

Terms used

Collecting lymphatics (lymphatic collectors, collectors, or collecting lymphatic vessels) are terms that include both, branching pre-collecting lymphatics (diameter approximately 30 -100 µm in humans, 43 (SD 15 µm) in mouse ear dermis) with endothelial lining supported by the BM (*tunica intima*) and lymphatic collectors (diameter range between 100 µm and 600 µm), which are additionally reinforced with layers of muscle cells and elastic fibers (*tunica media*), and loose connective tissue (*tunica adventitia*) that harbors blood capillaries of *vasa vasorum*[1]. Because the exact meaning of the term 'collecting lymphatics' depends on the context, here we refer to other than pre-collecting lymphatics as terminal collecting lymphatics that are located within appendages. The location determinant also distinguishes these collectors from the lymphatic trunks.

Migratory burst is the time a moving nematode spent on active migration (during its directional net migratory advances), i.e., in addition to head or tail, the central part of a nematode was translocated to a new position.

Migratory lag is the time a moving nematode spent in the tissue without net migratory advances (periods of apparent inactivity).

Maximum velocities (speeds) of migrating nematodes were calculated from the total distance that a nematode traveled during active migration (i.e., during migratory bursts) and calculated from a single regression of distance over time. As long as a nematode translocate most of its body to a new position, movements during migratory bursts were used to calculate nematodes velocities even when a nematode came back to its original 2D position during the move. These migratory bursts were distinguished from null velocities for backward-forward movement of the nematode head or tail. Therefore, vector velocities

equal their scalar speeds. Maximum velocities were used to compare filariae and nematodes migration speeds.

Average maximum velocities (speeds) are the maximum velocities averaged from directional migration of multiple nematodes (six soil-derived nematodes and four filariae migrating in the skin). These statistics depend on the variable time the nematode spent in the tissue on migratory lags, hence they were not used to compare speeds of filariae or nematodes.

Soil-derived nematodes is a polyphyletic grouping that includes free-living (bacterivores, fungivores, and predators) nematodes but also conditional zooparasites or zooparasites that have a transient parasitic phase in animals (e.g., *Strongyloides*). The protected location from which the terrestrial nematodes were probed minimized the risk of isolation of animal-derived parasitic nematodes. Similar to free-living nematodes, plant parasites spend varying portions of their life cycles in the soil environment and are the second to free-living bacterivores and fungivores most numerous group of nematodes found in the soil[2].

Replications

Primary lymphedema (Chy3/C3H mice). In two experiments we used all the Chy3-C3H mice that showed persistent lymphedema in the hind leg (n=4-5 each, mice were collected during 6 month breeding period).with equal numbers of C3H control mice. Each mouse was injected with 40 filariae.

Secondary lymphedema (surgery, C57BL/6) mice. Two experiments with the following groups: 3 mice in Control groups, 4 sham-operated and 4 and 6 in the second experiment in the lymphedema group. In the first experiment, one mouse was lost from the control group before the end of the experiment. In the lymphedema group, lymphatics in four (4) mice that underwent lymphatic occluding surgery but had no sustained change in tail diameter, were considered functional collecting lymphatics (four mice out of 10 mice in two experiments), therefore these mice were not used in the experiment. Additionally, two out

of 6 mice in the second experiment developed tail necrosis and were euthanized. Each mouse was injected with 40 filariae.

Due to lack of prior expectations that would warrant a test of the preliminary hypothesis of filariae and soil-derived nematodes behavior in the skin, the imaging of filariae and soil-nematodes migration in the skin was performed as an observational study, therefore the size of experimental groups could not be pre-determined[3]. Confocal microscopy imaging was performed on 14 mice injected with filariae, and 5 mice injected with soil-derived nematodes. Intravital imaging attempts were performed on 11 live-labeled ears injected with filariae, and 9 intact (unstained) ears injected with soil-derived nematodes. The imaging of subcutaneous spread of filariae was intended to verify whether fluorescent covalent labeling of filariae interferes with their *in vivo* migration behavior. The whole-body near-infrared imaging was performed twice. Each mouse was injected with 40-50 filariae.

The *in vitro* experiment that aimed at comparison of the effect of the flow on filariae migration was performed with the same on a single day, in the same maze chamber that was used alternately for static and flow tests with the following order of the tests: static (7 larvae), flow (2 larvae), static (2 larvae), flow (23 larvae), flow (7 larvae). On the other occasion, the static experiment was performed once (6 larvae), and the flow experiments were twice (with 5 larvae and second time, with approximately 45 larvae). The variation of larvae in these tests was the result of variable efficacy of filariae deposition in the Central Station. The tracking of larvae migration for the calculations of RNDs and filariae velocities was performed for runs best matching the number of larvae, that is, static and flow tests that had 7 larvae migrating within the maze of the chamber. Dying or dead larvae (larvae that moves frequency significantly differ from the rest of the group) were not included in the analysis. The movies of the other tests are available upon request.

The in vitro experiment showing filaria entrapment in the collagen gel was performed twice. The second movie showing entrapment of migratory larvae is available upon request.

Intradermal injection of nematodes (detailed)

Pipette tips, syringes, and needles that came into contact with nematodes were silanized with 2% dimethyldichlorosilane in 1,1,1-trichloroethane (Repel-Silane, GE Healthcare). After cleaning the needle-holding chamber with deionized water, the 29G needle was attached back into the syringe, and the residual air was removed with the mild push of the plunger. The plunger was then fixed in the position by tightening the plunger screw, and nematodes were allowed to accumulate at the needle inlet by hanging the syringe in the vertical position for 10 min before injection. Because the large size of nematodes (15x300 μm) cannot be forced into the dermis, the needle was first inserted 5 mm deep to create an intradermal pocket.

The primary (Chy3, hind paw) and secondary tail lymphedema models

The terminal collecting lymphatics within appendages (in mice: one in a limb, two in the tail) constitute a bottleneck for the entire afferent lymphatic drainage system from that appendage[1]. Hence, the occlusion of only these collectors blocks the drainage from the entire afferent lymphatic vasculature that is manifested with the tissue edema and fibrosis. These signs of occluded lymphatics can be used as guideposts for selection of experimental animals with functionally occluded lymphatics, which is the necessary condition to verify the absolute necessity of lymphatics for the systemic dissemination of filaria larvae.

To minimize the risk of compensatory adaptation of filaria to an abnormal skin environment, specific occlusion of collecting lymphatics (within appendages) with minimal change to the architecture of the dermal blood vasculature and dermal interstitium were the primary objectives of the experiment

Primary (Chy3) lymphedema induced by developmental occlusion of limb collector

A Chy3 genetic mouse model has a large chromosomal deletion that includes the VEGF-C gene, but it still develops a functional, though less dense, capillary and pre-collecting lymphatics in the skin[4]. In our specific-pathogen-free colony, this genotype had low penetrance (9 out of 370 mice, approximately 2%), with palpable primary edema in the lower paws as the main phenotypic manifestation of occluded limb collector in adult mice. The phenotypic manifestation of lymphedema and not the genetic screening was the criterion for designating mice in the control or lymphedema group. However, either because of incomplete occlusion of collectors (we cannot exclude the varying degree of phenotypic penetrance of the Chy3 genotype) or an unclogging effect of the injection[5], these experiments showed only correlative preference of L3 filarial larvae for the lymphatic route (as compared to the secondary lymphedema).

By comparison, the K14-VEGFR3 model is characterized by a complete absence of lymphatics in the dermis, yet they do not develop subcutaneous swelling[6]. Therefore, a complete lack of lymphatic capillaries might lead to compensatory changes in fluid distribution the dermis. Also, the absence of lymphatic capillaries (endothelium-secluded tissue voids), might change the tensile properties of the dermis[7], which could enable a compensatory but artificial mechanism by which filaria larvae enter into or migrate along blood vessels.

Secondary lymphedema model, induced by excision of lymphatic collectors

Developing an animal model of secondary lymphedema has been a subject of frustrations for over a century, and with few exceptions (models in dogs[8,9] and mice[10]) there is no reproducible animal model of the disease. The induction of mouse tail lymphedema by excision of skin with collecting lymphatics is a challenging procedure where a high number of individual mice (90% or more) had to be terminated before they develop signs of lymphedema due to tail necrosis, which is a consequence of wound infection and tissue ischemia, the effects of accidental cauterization of main blood vessels during the surgery. We have

optimized many aspects of this method that produced persistent edema in approximately 50% of mouse tails (an increase from 5-10% of the original method), that is, it led to the long-term doubling of the tail diameter distally to the surgery site. Since no cauterization was used to prevent bleeding during the operation, the clear view during the surgery was preserved by temporarily blocking the blood circulation in the tail with a rubber band tightened around the base of the tail. After the surgery, the rubber band was cut, allowing normal blood circulation in the tail, resulting in post-surgical bleeding from the wound. The excision of the skin fragment resulted in cessation of drainage in cutaneous capillary and subcutaneous collecting lymphatic vessels. Edema that persisted over the first 14 days, it became persistent and irreversible. However, without the cauterization and with the wound protective dressing, edema resolved spontaneously in 50% of cases one week after the surgery. Additionally, two out of 6 mice in the second experiment developed tail necrosis and were euthanized. This resulted in an uneven number of mice in the lymphedema and sham-control groups (the experiment was repeated once). Nevertheless, the modified tail lymphedema model that is specifically induced by complete cessation of lymphatic collecting vessels is the only feasible animal model where persistent tissue edema and tissue fibrosis act as a reliable sign of complete lymphatic closure[7].

Morphology-based identification of lymphatics vs. lymphatic (Prox1)-reporter mouse

Migration of filarial larvae in the skin was imaged in the living tissue with basement membranes stained for collagen IV. We wanted to avoid using the lineage-specific reporter mice, such as mice that express fluorescent tdTomato protein in lymphatic endothelium in Prox1 promotor-dependent fashion, as this approach restricts observation to the lymphatic system in the skin, and a prori deprive results from the information about blood vascular context of the tissue. Importantly, as we show, the basement membrane, and not the endothelial lining, could mechanically resist the parasite invasion but also prevents it from lumen escape.

In Supplementary Fig. 8, we also showed that due to incomplete penetrance of a tdTomato reporter protein, its expression in lymphatic endothelium is non-homogeneous, with lymphatic segments almost entirely deprived of Prox1-dependent accumulation of tdTomato reporter protein. Because of that, imaging of lymphatic injuries inflicted by invading nematodes could lead to the identification of false-positive sites of entry in lymphatic compartments with weak or no expression of reporter proteins.

It is important to note that tdTomato reporter protein occupies the visible and the least phototoxic channel that we used for imaging of TRITC-labelled nematodes. For that reason, we did not attempt to use reporter lymphatic mouse in free-nematode experiments as lymphatic reporter protein could mask the signal from weakly labeled smaller plant-parasitic nematodes.

Finally, fluorescent proteins are incompatible with the Murray clear (benzyl benzoate/benzyl alcohol-based clearing method), which is still the optimal clearing approach for preservation of morphological details in mechanically unprocessed tissues[11].

Statistical analysis (detailed)

Hence, the effect of primary lymphedema in Chy3 mice on filariae migration to the pleural cavity, the comparison of the diameter of soil-derived nematodes categorized according to their lymphatic localization, and the *in vitro* effect of the flow on the relative net displacement (comparison between static ($\overline{Rf}_{\text{stat}}$) and flow ($\overline{Rf}_{\text{flow}}$) were tested with one-tailed Mann-Whitney U-test using values from two pooled experiments. The velocities of migrating filariae and soil-derived nematodes were manually tracked in Fiji (ImageJ).

Microscopy equipment and settings

Intravital epifluorescence imaging

Intravital imaging was done with M250 FA (Leica Microsystems CMS GmbH, Wetzlar, Germany) motorized fluorescence stereomicroscope, equipped with 1× lens (linear system magnification from 7.5× to 160×) or 2× lens (linear system magnification from 15.6× to 320×) and DFC 350 FX camera controlled by LAS AF 2.9 software (Leica Microsystems). The exposition of 12-bit images was adjusted to fill the dynamic range of an image. For fluorescence imaging, the range of camera grey-scale values was set from 0% (minimum) to 5.1% (maximum) and set gamma correction to 1. With gain set to minimum (1) and a maximum power of the Leica fluorescence light source (EL6000), the exposure time was adjusted to fill the 12-bit dynamic range without clipping highlights, midtones, and shadows, and was between 0.2 and 2 seconds. The camera frame rate was 20 MHz with image resolution 1000x1200 pixels. Original Leica lif files are available upon request.

Optical sectioning of a thick whole-mount preparation of cleared tissue

Leica SP5 confocal microscope

Confocal images in Figures 1-3 and Supplementary Figures 2-4 were taken with Leica SP5 inverted confocal microscope equipped with beam-splitting prisms and the White Laser light source tunable from 470nm to 670nm in 1nm increments. 12-bit images were collected at 1012 x 1024 resolution with HC PL APO correction ring 20x/0.75 immersion lens or HC PL APO 63x/1.40 oil objectives. At optimal (following the Nyquist theorem) Z step (0.8 µm for the 20x lens and 0.25 µm for the 63x lens), XY planes were scanned with the resonance scanner speed of 8000 HZ, each field was averaged 48 times, and the signal was collected simultaneously by two HyD detectors. Images were processed with Imaris 7.4 software (Bitplane) and stored as Imaris (.ims) files (available upon request).

Olympus spinning disk microscope

As marked in the text, high-resolution images in Figure 5 and Supplementary Figure 5 were collected with Olympus IX inverted confocal microscope equipped with Spectra X (Lumenocor) LED light engine® and ORCA-Flash 4.0 sCMOS camera. 16-bit images (2048 x 2048) were collected with UPLSAPO 20XO 20X/0.85 or PLAPON 60XO 60X/1.42 oil objectives. The exposition of 16-bit images was adjusted to fill the dynamic image range unless the exposition was longer than 3 seconds. At optimal (following the Nyquist theorem) Z step (0.65 µm for the 20x lens and 0.2 µm for the 60x lens) snapshots of XY planes were taken with the 0.5 to 3 seconds exposition without signal averaging. Images were processed with Imaris 7.4 software (Bitplane) and stored as Imaris (.ims) files (available upon request).

Image processing

Diameters of lymphatic capillaries, collectors, filaria larvae and nematodes were measured in zinc-fixed and cleared tissues, which cancel the effect of tissue processing artifacts in reciprocal comparisons.

Images from the confocal Leica microscope and spinning disk microscope are referred to as confocal images. Images from the Leica microscope were obtained with the scanning technique, therefore, they are a circle on the black background of the square-shaped. Images from the Olympus microscope are taken with the camera, hence they reflect the natural roundness of optical systems. An additional consequence of collecting signal simultaneously by all phototubes of the camera is the bell-shape background amplification in the center of the image. This is a result of the additive accumulation of photons from the nearby spots in the imaging plane. To compensate for that 'bell' effect, images from the Olympus were processed with a current version of Fiji (ImageJ) software using a 'Subtract background' option and 'Rolling ball' setting set to 100.

Further image processing of fluorescent channels was done in Imaris software by adjusting the minimum and maximum of image extended dynamic range (12 bit for Leica, 16 bit for Olympus). Unless explicitly

noted in the text, the confocal images are presented as maximum intensity projections (MIP) or MIP of the defined optical sections (generated with Ortho Slicer or Clipping plane tools of Imaris). The thickness of XY, XZ or YZ optical cross-sections is indicated in the figures.

The image processing aimed to preserve shadows and midtones and emphasized tissue structure. Gamma correction was generally avoided and was set to 1. However, when structures (image highlights) or background (shadows) were lost in the adjustment of maximum or minimum range, the gamma was corrected within +/- 0.25 range. Channel-based surfaces (Surface tool in Imaris) that serve to emphasize particular tissue structures irrespectively of their fluorescence intensity (signal variation) are marked in the figures.

Additionally, in situations when strongly labeled structured would have masked another part of the image, the mask was used to enhance the weak signal locally. This procedure was performed for bacterivorous nematodes in Figure 5 B-D, which mouth part was approximately labeled 10 000-times stronger than the nematode cuticle. To reveal the shape of the entire nematode in such an extreme situation, the surface mask was created over the body except for the highly fluorescent stoma and pharynx, and the intensity of those parts of the nematode was increased over the mouth part. Other than that, no touch-up or local enhancement tools were used during image processing.

Processing of intravital microscope images was done in Leica LAS AF 2.9 by adjusting the minimum and maximum of the 12-bit image. Images were exported in Jpg (for the generation of movies) or Tif for figure assembly). When possible (when sequence size was smaller than 500 Mbit), videos were generated directly from LAD AF software. Except for **Supplementary movie 1**, only basic, single color lookup tables, that is, red, white, green or cyan with fixed Hue and Saturation) were used in the images to represent the fluorescent signal intensity, where the increase of the signal is reflected by the linear increase in the color Brightness (Value).

Supplementary notes

Supplementary Note 1

The absolute requirement of lymphatics by non-lymphatic *L. sigmodontis* filaria

The absolute requirement for functional lymphatic circulation that excludes the involvement of blood vessels in completing the migratory route of Litomosoides sigmodontis filaria has not extended beyond the empirical correlations, that is, the fact that L. sigmodontis infective larvae use lymphatics during their migratory phase. For example, in the most recent report, Karadjian et al[12] present the indication that *L. sigmodontis* prefer lymphatics for their migration to the lymph nodes. However, the correlative design of the experiment shown in the Supplementary Figure 1 in "*Migratory phase of Litomosoides sigmodontis filarial infective larvae*" cannot unequivocally exclude the potential contribution of blood vessels in delivering filariae to the lungs. Hence, this experiment, as many reports before[13,14,15], presents a correlation that supports the hypothesis where *L. sigmodontis* larvae prefer the lymphatic route but do not exclude the potential contribution of the alternative blood vascular route.

Supplementary note 2

Non-lymphatic *L. sigmodontis* to verify filariae preference for lymphatic route

Despite that *L. sigmodontis* has been used as a model of lymphatic filariasis[16], it is not a lymphatic filaria. This is because its adults, in contrast to human lymphatic filariae, that is, *Wuchereria bancrofti* or *Brugia* species, are most frequently found in the pleural cavity and not in lymphatic collectors or lymph nodes. Therefore, *L. sigmodontis* infection cannot, even on a theoretical level, lead to pathologies that are the result of human lymphatic filaria infections. Contrary, *L. sigmodontis* infection resembles an asymptomatic human mansonelliasis caused by *Mansonella perstans*, which adults live in pleural and peritoneal cavities.

Why was it necessary for us to show that *L. sigmodontis*, a non-lymphatic filaria exclusively requires lymphatics for establishing the patent infection? Populating lungs from the skin can be more directly achieved by the initial invasion of skin blood vessels instead of the indirect path through the lymphatic circulation, which ends up in terminal lymphatic trunks draining lymph to subclavian veins. Despite that, we found here that the via lymphatica, the more convoluted thus less effective path, is exclusively used by *L. sigmodontis* infective larvae as a route to reach the pleural cavity. This suggested that the advantages of using the lymphatic route outweighs its costs. As such, it was likely that a decision to invade or avoid lymphatic entry may also determine the migratory fate and habitat selection of other filaria species.

Supplementary Note 3

Identification of organs within labeled filaria helped to established filariae polarity

The digestive tract begins with the mouth opening located at the tip of the larva and ends with the anus valve approximately 9/10 down its length. A nerve ring surrounded the pharynx at the anterior end of the animal was located in front of the asymmetric excretory pore.

Supplementary Note 4

Ear skin dermis is ideal for imaging but not for the inoculation of nematodes

The use of the thin ear skin for the inoculation of nematodes has some significant drawbacks. For example, due to the lack of subcutaneous space, only the intradermal injection can be performed in the mouse ear dermis. However, distribution by injection of large particles, particularly millimeter-long nematodes, is blocked by the resistance of the interstitium. The dense extracellular matrix acts as sieve allowing only solute and small particles to be injected beyond the limits of the tissue pocket created by the needle. Because of this sieve effect of the dermis, larvae can only be deposited within a pocket pre-formed by an injecting needle. Additionally, the skin of the mouse ear is atypical as compared with skin elsewhere in

the mouse body, it is immobilized to subcutaneous muscles and cartilage. Together with the higher stiffness that results from the absence of hypodermis, the skin of the ear does not readily contract after the injury unless it is completely detached from the underlying structures[17].

The histology of the ear skin complicates the intradermal inoculation of nematodes as the post-needle dermal pocket does not close after the needle is retracted. Therefore, inoculated nematodes are left entrapped in the fluid-filled pocket made by the injection needle, with only a few being able to escape into the dermis. We pictured this situation in **Supplementary movies 3** that show the entrapment of filaria and nematodes within the fluid pocket of the collagen gel. From the above one can correctly infer that the ear is the least optimal nematode inoculation site. However, we did not intend to create the optimal method for parasite inoculation; we merely wanted to adopt our imaging method for visualization of in-tissue migration and lymphatic entry of nematodes. Therefore, even though the ear is the least optimal dermal inoculation site, it is the only location that can be used for the imaging experiments using our rapid intravital imaging system. As a side note, it is worth noting that the more effective, subcutaneous injection of filaria larvae, which is the standard way of filaria inoculation in animal models, is not a natural way in which filariae are transferred to the host as larvae cannot be directly deposited in a subcutaneous space by an arthropod, such as mosquito or fly.

Supplementary Note 5

Morphology of collecting lymphatics is revealed by their basement membranes

Collagen IV-labeled basement membranes revealed the morphology of tissue stationery (and non-hematopoietic) structures present in the skin, including nerves, blood, and lymphatic collecting vessels, striated muscles and adipocytes[7,18,19]. Lymphatic morphology is unique and characterized by the uneven diameter of the vessels along its length, which is a consequence of the presence of flow-resistant post-valve sinuses, which are extended backward as oppose to valves in the elastic veins[1]. Additionally,

lymphatic collectors in the mouse are marked by scare and incomplete imprints of smooth muscle cells that enwrapped the continuous basement membrane tube of lymphatic collector[7,18]. The presence of intralymphatic valves, sparse smooth muscle cells, and a thick basement membrane also distinguish pre-collectors from blunt-ended, usually Lyve1-positive capillary lymphatic[17].

Supplementary Note 6

Negligible lymph flow in anesthetized animals is restored by intradermal injection

Sleeping and anesthetized animals have negligible lymph flow in collecting lymphatics[20,21]. However, the flow within lymphatics can be artificially restored with the intradermal injection of fluid. This temporarily produces visible through the skin injection-dependent edema that locally forced solutes and solvent into lymphatics. As the edema plateau within minutes after the injection, the orientation of larvae at the valves should reflect the static effect of valve morphology (if the valve morphology was a relevant factor). Therefore, in the absence of potential lymph flow guidance, larvae should be less frequently located at the valve trunk (afferent side of the valve) as their valve opening can be more frequently localized and crossed from the lymphatic trunk. Contrary, larva migrating in the opposite to lymph node direction, should have a less chance of localizing and crossing the valve opening from the efferent side of the valve as valve opening within the sinus of the lymphatic valve occupies a lower fraction of the sinus surface. Further, since larvae can break on through the lymphatic wall, the closed state of the valves during minimal lymph (i.e., during animal sleep), should not pose a significant barrier for larva migrating in any direction.

Supplementary Note 7

Epifluorescence microscopy allows prolonged intravital imaging

The immune staining and the intravital epifluorescence imaging can be performed in the thin (two-dimensional) tissues, such as the thin dermis of the ear skin, which alleviate the need for optical sectioning and by this, the use of high power (laser) light source. This has a consequence for the choice of fluorophores used in immunolabelling. Due to the spatial separation of chromophore centers, fluorescent proteins are photostable and produce minimal levels of free-radicals. This is in contrast to small-molecular fluorophores that are prone to quenching but more importantly, are photo-reactive with the surrounding biomolecules. The epifluorescent light source allows fast, prolonged imaging of sequentially exposed fluorophores at multiple fields of view, which was ideal for capturing rare events such as lymphatic entry or intra-lymphatic migration. Additionally, extracellular labeling of the live tissue provides an opportunity to protect it from phototoxicity by applying a high concentration of isotonic antioxidant, prepared by replacing chloride anions of Hartman's solution with 70 mM ascorbate ions, which almost entirely eliminates the problem of fluorophore quenching and phototoxicity[18,19].

The alternatives to epifluorescence imaging, the multi-photon microscopy, allows imaging the intact skin but due to necessity of XYZ scanning, the slow imaging practically limits its application to a single tissue location that can be imaged at a single resolution (20x lens, NA 1.0), restricting the output of the technique to statistical probing analysis. Also, as the high power lasers (orders of magnitude stronger even considering the required two-photon absorption) burst generate free-radicals in non-protein fluorophores, the imaging would likely result in the rapid death and the destruction of fluorophores in TRITC-labeled nematodes[19]. Similar limitations can be ascribed to confocal or spinning disk imaging techniques.

Supplementary Note 8

Filaria in the overlaid fluid could not enter the dermis

After partially exposed dorsal skin was pre-stained for collagen IV, larvae were injected into the distal fragment of dorsal skin so that the entire injection site was protected by the cartilage and intact fragments of the ventral and dorsal skin flaps (Supplementary Fig. 2A). Larvae that left the inoculation pocket entered the unstained dorsal skin and in some cases continued migration within the stained dermis (**Supplementary movie 4**). Some larvae left the exposed dermis after migrating perpendicularly towards the surface of the stained skin. Once larvae were in the buffer, they could never re-enter the dermis.

Supplementary Note 9

Transient injection-driven lymph flow replace physiological interstitial currents

The physiologically-driven lymph currents in anesthetized animals are negligible[21,22] thus they cannot be responsible for directed filariae migration in the experimental animal. However, the imaging of larva shown in **Supplementary movie 9** started 3 minutes after larvae inoculation. Therefore it is probable that its directional movement could be driven by the lymph flow initiated with the intradermal injection and subsequent injection-driven edema[7,20]. However, the long-distance nematode traveler shown in Movies 16A and 16B, started its migration over 30 minutes after the nematodes were injected, which is long after the injection edema pressure equilibrated with the surrounding dermis (injection edema in the skin can be seen for up to 10 minutes after the intradermal injection).

Supplementary Note 10

Velocities in lymphatics explain fast filaria relocation to lymph nodes

Traversing the 3.5-cm distance between the inoculation site and the draining lymph node[17] at the maximum in-lymphatic speed of 731 µm/min should take approximately 5 minutes. However, for up to

three hours after the inoculation, 15 larvae were found only in the subcapsular sinus of the lymph node (Supplementary Fig. 3B), suggesting that channels within the lymph node, at least temporarily, impeded the further movement of the *L. sigmodontis* larvae. This is in agreement with the clinical observations showing that the lymphatic filariae *Brugia* species and *W. bancrofti* are most frequently localized in the afferent vessels near or within lymph nodes[23].

Supplementary Note 11

Detailed analysis of the collector breach (filaria path from Movie 9).

The ease of relocating CD31 but not VE-cadherin is understandable because only VE-cadherin is essential for maintaining the integrity of the endothelium forming adherens junctions between endothelial cells[24], while CD31 is dispensable for zipper junction formation in collectors[25].

The variable levels of CD31 at the anterior and posterior sites of the lymphatic breach also excluded a potential handling artifact because changes in live cells could only occur prior to tissue fixation and subsequent mechanical separation of the dorsal and ventral skin fragments. The pre-collector wound formed a large, irregular, but incomplete breach in the lymphatic endothelium and the supporting basement membrane. The detachment of the lymphatic endothelium overlapped entirely with the loss of basement membrane, with a higher cell density apparent on the afferent side of the collector's breach in the maximum intensity projection (MIP) image (Fig. 3 Bi). A single-plane (160 nm) cross-section from the central plane of the afferent pre-collector segment showed that the higher endothelial cell density resulted from the relocation of the fragment of collector wall towards its valve (Fig. 3 Bii). The relocated and folded collector basement membrane had the same thickness as the remaining vessel walls and was still supported by a single layer of endothelial cells.

Supplementary Note 12

Velocity of fluid flow within a microfluidic maze

During the steady-state, the lymph flow within skin lymphatics range between 1 and 20 µm/s, but it can be subjected to extreme fluctuation, even by orders of magnitude, in a physically active animal[22], during inflammation[26] or during sleep[21,22]. To account for these factors, we decided to exceed the physiological range of lymph velocities and diversify the *in vitro* currents between 1 and 50 µm/s. Using the iterative computations of the flow velocities in the maze, we found that the 15 nL/s inlet flow rate from the NE port was required to fulfill these requirements.

Supplementary Note 13

Verification of the mechanism of filaria invasion with non-zooparasitic nematodes

The purpose of injecting random species of soil-derived (mostly, free-living and phytoparasitic) nematodes was to show that even without adaptation for parasitic life style, nematodes from different trophic groups are able to migrate in the tissue and enter lymphatics. This, in turn, would indicate that skin migration and lymphatic invasion is a symplesiomorphic trait that exists at least in all descendants of the common ancestor of tested nematodes.

In contrast, experimental iterations that link a tissue homing mechanism with successive species of filariae would not allow any generalization of the mechanism found with *L. sigmodontis* even over the filaria group itself. Similar, validation of the *L. sigmodontis* mechanism in other groups of non-filarial nematode parasites, could not exclude the possibility of phenotypical convergences, which are the expected (and in fact frequent) responses to similar selective pressures. However, showing that the abilities responsible for tissue wondering and lymphatic break-in are shared with non-parasitic nematodes from within the same crown group (clade) would indicate that the trait was not directly derived as the response to

parasitic live style, and as a plesiomorphy, it had to be shared with all nematode within the clad, that is, groups of sister taxa linked by their common ancestor. Therefore, instead of repeating experiments performed on *L. sigmodontis* in, for example, *Brugia* or *Dirofilaria* models, we asked if nematodes that belong to vastly different trophic groups (bacterivores, plant parasites), share the *L. sigmodontis* traits that allow them to migrate in the skin and invade lymphatics. If so, these experiments would indicate that the tissue migration and lymphatic invasion traits are the ancestral traits of free-living, phytoparasitic and filaria groups and must also belong to their sister taxa.

Further, we specifically avoided using known skin-invading parasitic models as any parasitic nematodes had to be suspected of carrying specific adaptations for lymphatic invasion and systemic spread. The free-living nematodes and plant parasites can be considered as attenuates zoo-parasites that are deprived of any of the potential (vertebrate) parasite-specific abilities, hence they are an ideal control that could exclude a potential specialization towards tissue migration and lymphatic invasion. Contrary, in the established mouse models of filariases, for example, the mouse model of *Brugia* infection, the expected results would merely duplicate the *L. sigmodontis* experiments as tissue migration and lymphatic entry of *Brugia malayi*, even accomplished with different kinetics, could not rule out the adaptive parasitic mechanism of the nematode dissemination.

Supplementary Note 14

Isolation of non-zooparasitic nematodes from soil samples

To minimize the risk of the isolating soil-derived zooparasites, soil samples were collected from a monitored park location not visited by wild, household or farm animals. Meanwhile, the Baermann funnel technique allowed collection of alive and actively migrating nematodes of various developmental stages and dimensions (**Supplementary movie 13**).

Supplementary Note 15

Technical challenges with intradermal inoculation of soil-derived nematodes

As opposed to subcutaneous delivery, intradermal injection of large particles such as filarial larvae led to their entrapment within a dermal pocket made by the insertion needle, with only a few larvae able to leave the injection site (see the filarial injection site in **Supplementary movie 1** and nematodes injection site in **Supplementary movie 14**). This intradermal entrapment became a severe problem when nematodes of various sizes and movement characteristics were injected in the post-needle intradermal pocket. Within the dermis, larger roundworms forced the smaller nematodes towards the surface of the cartilage, causing their release onto the exposed skin. However, without the surgical exposition of the dorsal dermis, we were unable to stain basement membranes for collagen IV, neither we could counteract the imaging phototoxicity with topical application of sodium ascorbate.

Supplementary Note 16

In-lymphatic migratory characteristic

In-tissue movements of filariae larvae and terrestrial nematodes have similar discrete phases, that is the movement with no net advance (Lags) and the fast, in-lymphatic-like migration at 635 µm/min. However, assuming that the nematode migration through the dermis occurred within lymphatics, we could not explain the nature of the third type of movement of the nematode, the in-dermis-like (132 µm/min) unidirectional migration. Its velocity was similar to the speed of in-dermis migration of soil-derived nematodes. However, the delayed in-lymphatic migration could be caused by frictions between nematode and the lymphatic wall that results from a mismatch between the lymphatic diameter and the girth and length of nematodes that are not adapted to skin parasitism.

Supplementary Note 17

Diameter-dependent distribution of nematode sin lymphatics

Below is the graphic presentation of data presented in the Results. Three nematodes (mean diameter 9.7 (SD 2.8 µm)) located entirely within collectors (two are shown in Fig. 5B-C and 5E) and three others (diameter 15.4 (SD 9.7 µm)) partially within collectors (one is shown in Fig. 5D), and 20 outside the vessels. These remaining 20 nematodes (diameter 19.6 (SD 9.4 µm) were located outside the inoculation pocket and outside basement membrane structures within the skin, that is, none were found even partially embedded within blood vessels. Out of the six nematodes that partially or entirely entered collecting lymphatics, five had the stoma and pharynx characteristic for bacterivores, and one was a non-feeding plant parasite with a characteristic stylet[2] within its mouthpart (Fig. 5E).

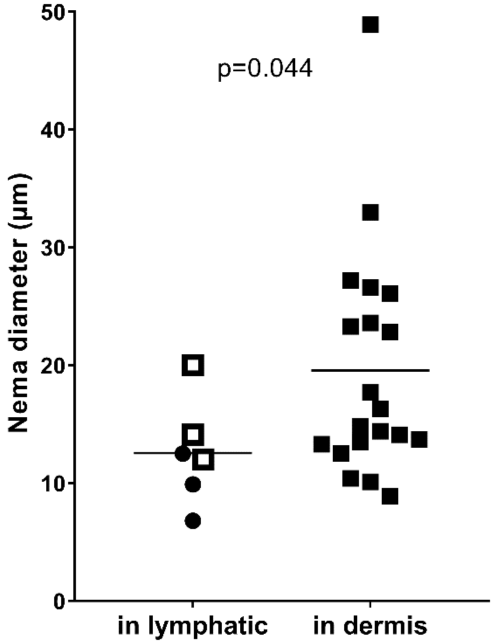

Nematodes entirely within lymphatics (black circles); nematodes partially within lymphatics (black–bordered white squares) lymphatics; nematodes that managed to enter dermis from the injection pocket

but were outside BM structures (black squares represent). Groups were compared with the Mann-Whitney test. Source data available as Source data file.

Supplementary note 18

Soil-derived nematodes are resistant to zinc-fixative

Soil-derived nematodes are highly resistant to zinc-fixation, even when the fixer was supplemented with 5% DMSO, 1% Triton-X-100 and 10 µg/ml pancuronium bromide at 4°C, nematodes were alive on the next day (some moving roundworms could be found in the skin 7 days later). However, submerging the tissue in zinc solution (original or modified as above) initiated their rapid escape from the tissue. Because of that, we were unable to apply our whole-mount staining protocol to the lymph nodes as in contrast to nematodes in the ear dermis, nematodes in the lymph node could not be directly accessed and rapidly killed with the application of 1% sodium azide. This is because long diffusion times in large chunk of LN tissue requires long incubations between changes of washing solution. The extensive washing is required when changing between zinc and azide salts as otherwise, they would form shock-sensitive zinc azide. For that reason, sodium azide can be only applied after extensive washing of the exposed thin tissue, such as dorsal dermis of the ear. For the same reasons azide could not be mixed directly with zinc fixative.

Supplementary Discussion

Although understanding the cues that govern the host identification is invaluable from preventive and epidemiological perspectives, this information cannot explain migratory decisions of a helminth once the parasite enters host tissues. For example, the finding that *Strongyloides stercoralis* detects urocanic acid, the first identified host-specific chemoattractant that helps to locate human skin, does not explain the convoluted routes used by this parasite within a host[27].

Owing to the lack of substantial experimental evidence, even minor indications are used to support daring but somewhat anecdotal hypotheses. For example, the in vitro secretion of metalloproteinases by infectious larvae of *Brugia malayi*[28] led to a generalized presumption that proteases are critical in filaria infection by allowing L3 larvae to penetrate through the tissue and migrate to the local lymph node[29,30,31]. Our imaging experiments suggested otherwise; instead of continued movement through the extravascular matrix, as would be expected if in-tissue migration depended on the continuous degradation of homogeneous interstitium of the dermis, filarial larvae, but also non-parasitic, soil-derived nematodes, moved through the skin with sudden and rapid surges, during which they could change or reverse the direction of their migration.

Similarly, it has been proposed that host proteins can chemotactically guide parasitic nematodes within the host[32]. The complex chemokine gradients within a vertebrate body provide a specific guidance system for various migratory cell types[33] and can be abused by microscopic viral or bacterial parasites. In contrast, nematodes respond to physicochemical gradients, such as chemicals (chemoreception), touch (mechanoreception), and temperature (thermoreception)[34]. However, nematodes cannot directly rely on hijacking the host chemokine-receptor systems as this mechanism, that is, the G-coupled peptide receptors is an evolutionary acquisition of animals from the phylum *Chordata* to which vertebrates belong[35]. Furthermore, because various filarial groups can independently colonize different vertebrate species[36] or even jump between unrelated hosts of different classes such as birds and mammals[37], it is unlikely that species-specific chemoattraction could be acquired multiple times (vertebrate parasitism developed five times in nematodes) through convergent evolution incidents that independently could re-develop chemokine receptor system[38]. On the other hand, lateral gene transfer could be the most profitable event for a filarial colonizer, allowing it to immediately gain access to host-specific functional receptors and hijack its guidance system; however, neither such transfer events have been shown for vertebrate parasites[38,39,40], nor the presence of chemokine receptors has been documented in

nematodes[35]. Finally, adaptation of system that is evolutionary costly (that links a parasite with its host) and technically challenging (that allow receptors to access chemokines while filtrating away the host defenses, e.g., proteases) over the universal mechanism of stochastic migration and vessel break-in entry, would have to be counter-balanced with selective advantages that are difficult to devise.

Instead, the distinction of various migratory routes within the host might rely upon secondary traits that for instance, limit the access of larvae towards or migration within lymphatics. However, due to the lack of appendages and circular muscles, nematodes have little room for modification. This anatomical simplicity restricts their movements to two main types: head searching and backward-directed waves. The phase and the amplitude of the movement waves depend on the environment (resistance) in which the nematode migrate[41]. In consequence, infective larvae of a parasite (or a migratory form of any nematode) switch its movement characteristics between environments in a predictable way[42]. In **Supplementary movie 3,** a filaria larva that is entrapped within gel pocket moved at a lower frequency and higher amplitude than the second filaria that was advancing through the gel. The difference of movement characteristic can be explained by the Wallace model of nematode movement[43,44], where frequency and amplitude are linked by a constant power output of the worm, which is specific for the nematode species and developmental stage. Hence, as the nematode must increase the force required to move within denser environments, it acts as automatic hammer drill by increasing the frequency of its waves and at the same time, reducing their amplitude so that its energetical balance remains unchanged.

The selection of the migratory route within the host might primary rely on gross anatomical modifications, such as changes in larval diameter or mouthpart shape, which restrict their entry into lymphatics, and the time the infective larvae spend migrating before the subsequent molt lock them in their current location. Consequently, even though lymphatics offer the only available (for brute-force invasion) escape path from the skin, controlling the time a larva spent in the given size provides the opportunity for the diversification of the dissemination routes by the differential termination of exit routes during larval migration, with a

mechanism similar to anatomical/mechanical entrapment of tumor metastasis[45]. Hence, the larvae entrapment can take place in the dermis or subcutaneous tissue, lymphatics, primary (pleural) or secondary capillary plexus allowing different species of parasites to establish their distinct habitats. For example, the infective larvae of *Loa loa* has the largest diameter (35 to 45 µm[46,47]) of all human filarial L3 larvae (15 to 25 µm[48,49]), which might allow *L. loa* larvae to fit into the wider of skin pre-collecting lymphatics (in human skin their diameter ranges between 30 and 100 µm[1]) with additional room for free movement that would allow in-lymphatic migration. Since the density of larger pre-collectors decreases exponentially with the increase of their diameter, most *L. loa* (72% in the mouse model[46]), that could fit only in the largest of mouse cutaneous pre-collectors (43 (SD 15 µm)), unsurprisingly remain in the subcutaneous space. In contrast, the small diameter of *Onchocerca volvulus* L3 larvae (20 µm) coincides with the shortest among all human filariae (2-3 days) time before molting to the L4 stage (as compared to 8 to 9 days for human lymphatic filariae). This might explain why *O. volvulus* usually forms nodules already within the wall of subcutaneous lymphatics[49]. Conversely, the fact that adults of the smallest human filaria, *Mansonella streptocerca* (L3 data is not available; after two molts adults reach the size of *L. loa* L3 larvae)[23]*,* are found in the skin, might indicate that their L3 larvae are too small (thus too weak) to migrate through the dermis or cross collector wall. As a side note, the difficulties in finding these basic anatomical parameters of L3 infective larvae, as opposed to mature filaria or their microfilaria larvae, indicates that future advanced experimentation must be paralleled with systematic basic morphometry of all larval stages of human filariae.

In contrast to chemokine-specific tropism, universal in vertebrate tissues fluid currents offer a non-specific guiding system, that could evolve even before adaptation to parasitic life-style. Using the microfluidic maze, we showed that filaria larvae exhibit a positive current tropism that might guide L3 larvae away from blood vessels, during lymphatic entry, and later during in-lymphatic migration. We did not attempt to verify these results *in vivo* due to a lack of an animal model in which interstitial fluid flow and lymph

transport can be experimentally controlled or even maintained at the physiological levels[22]. This problem is particularly evident in anesthetized animals whose lymphatic transport from the peripheral tissues can drop by two orders of magnitude[20,21,22]. Importantly, intradermal injection of fluid is known to cause a transient rise in the magnitude of the hydrostatic pressure of the interstitial fluid, which forces the flow of the lymph[17,20]. This temporarily reinstated directional fluid currents might guide the migration of larvae to lymph nodes within minutes post-inoculation. However, since most of our intravital imaging experiments had to be performed in animals under anesthesia, we assumed that directional fluid currents (post the plateau of injection-driven lymph flow) had a negligible effect on larval migration.

As the lymph flow drop during sleep or animal rest is a physiological process, we suspected that there might be an auxiliary mechanism that could assist in larvae unidirectional movement within lymphatics. Lymphatic collectors, maintain unidirectional fluid flow due to the morphology of funnel (a single cuspid valve of the pre-collector lymphatics) or bicuspid intralymphatic valves (pre-collectors and collector lymphatics) that close when a retrograde flux of the lymph presses on the walls of lymphatic sinuses and valves in the efferent collecting vessel[7]. However, in contrast to the unidirectional flow of passive lymph[7,50], actively moving parasitic nematodes cannot rely on differences in hydrostatic pressure. Instead, they can take advantage of the valve morphology, which assures a higher probability of crossing the valve in the efferent direction towards the lymph node.

Our rapid intravital epifluorescence imaging system allows capturing nearly any event that takes place within the entire ear dermis at a microscopic level, hence, it is designed to capture rare, qualitative events. However, due to their scarcity, calculating the inferential statistics was not always possible. For example, the observations of fast in-lymphatic migration of filaria larva and soil-derived nematode could on its own, only provide an outlook of the in-lymphatic migration. However, identification of the site of entry of the filaria migrating within a lymphatics revealed that the entry process could heavily damage the wall of the collector. This observation focused our attention on lymphatic injury sites located proximal to the

nematode, which otherwise would likely have been disregarded as preparation artifacts. Further, even though the migration of filaria larva and soil-derived nematode within lymphatic collectors were single events, their velocities were similarly a five to six times the speed of the average in-dermis migration of respected, filaria larvae or soil-derived nematodes. Because the velocities of *in vivo* migrating nematodes fall into discrete values, we conclude that also the *in vitro* mean velocity of filariae migrating within the maze might be representative of the in-lymphatic movement of the filaria larva. *In vitro*, the magnitude of filariae velocity was not affected by fluid currents, despite the presence of even 10-times faster fluid flow. This emphasized the larvae capability for handling the channel (and likely also lymphatic) architecture to their advantage. However, the active migration within pre-collectors does not contradict the possibility that parasitic nematodes could be passively carried with the lymph in larger lymphatic collators or lymphatic trunks (terminal lymphatic collecting vessels).

Nevertheless, these experiments helped to eliminate from the further analysis less probable hypotheses, such as protease-assisted non-migratory fusion of larvae with lymphatic vessels or passive proteolytic passage of larvae through the skin. In fact, due to a lack of recorded in-tissue and in-lymphatic velocities or migration patterns, any hypothetical model could have been equally valid. Therefore, despite the limited number of recorded migratory events, the initial observations directed a search for further supporting evidence using optical-sectioning confocal imaging of whole-mounted skin preparations.

This combination of imaging tools minimizes the probing approach and can be directly applied for the analysis of the infective stage of any dermal parasite, and with modifications, for intestinal and likely ectoparasites[51]. The confocal imaging was made possible with a protocol that allows intravital labeling of nematodes and the adapted staining procedure. The location of labeled filariae buried within the tissue was initially approximated with low magnification techniques, and later, the pre-selected field was imaged with a high-resolution confocal microscope.

Interestingly, the rates of active filarial migration in lymphatics are of the highest directional migration velocities recorded in the vertebrate tissue and are 5-6 times the speed of the fastest unicellular organism, malaria *Plasmodium* that migrates in the dermis at the average rate of 100 µm/min[52]. On the other hand, the rates of nematodes during the cutaneous burst of in-tissue migration are 5 times the speed of the in-tissue migration of the fastest migrating vertebrate cells, that is, the chemokine-guided migration of activated neutrophils (25-30 µm/min[18]).

Instead of isolating specific species of non-zooparasitic nematodes we focused on feeding behavior associated with a free-living or phytoparasitic lifestyle, which ensured that isolated nematodes could not acquire convergent zoo-parasitic adaptations. In consequence, the fact that *L. sigmodontis* filaria (suborder *Spirulina*), bacterivores (suborder *Rhabditina*), and plant parasites (likely suborder *Tylenchina*) migrated in the dermis and within lymphatics indicated that tissue migration and lymphatic entry are symplesiomorphic characteristics of these groups and their ancestral *Chromadoria* subclass. *Chromadoria* is the largest monophyletic clad within *Nematoda* phylum that includes the majority of free-living terrestrial nematodes, that is, bacterivores, fungivores, herbivores, algivores, omnivores, and predators, and also the vast majority of vertebrate, insect and plant parasites. Large nematode predators and intestinal parasites such *Trichinella spiralis* belong to the *Dorylaimia* subclass*,* while marine and freshwater free-living and phytoparasitic nematodes are members of *Enoplia* subclass[2,38,53].

References

1. Földi M, Földi E. Structural elements of the lymphatic system. In: *Földi's textbook of lymphology*. 3rd. Elsevier GmbH (2012).

2. Bernard EC, Noe JP. Plant-parasitic nematodes. In: *Plant pathology. Concepts and laboratory exercises* (Ownley BH, Trigiano RN). 3rd. Taylor & Francis Group (2017).

3. Observational study (syn: Nonexperimental study). In: *A dictionary of epidemiology* (ed^(eds Porta M). Oxford University Press (2016).

4. Dellinger MT, Hunter RJ, Bernas MJ, Witte MH, Erickson RP. Chy-3 mice are VEGF-C haploinsufficient and exhibit defective dermal superficial to deep lymphatic transition and dermal lymphatic hypoplasia. *Dev. Dyn.* **236**, 2346-2355 (2007).

5. Kilarski WW, Herbert J, Bikfalvi A. Methods for mapping the extracellular and membrane proteome in the avian embryo, and identification of putative vascular targets or endothelial genes. *Methods Mol Biol* **1722**, 31-56 (2018).

6. Makinen T*, et al.* Inhibition of lymphangiogenesis with resulting lymphedema in transgenic mice expressing soluble VEGF Receptor-3. *Nat. Med.* **7**, 199-205 (2001).

7. Kilarski WW. Physiological perspective on therapies of lymphatic vessels. *Adv Wound Care (New Rochelle)* **7**, 189-208 (2018).

8. Drinker CK, Field ME, Homans J. The experimental production of edema and elephantiasis as a result of lymphatic obstruction. *Am J Physiol* **108**, 509-520 (1934).

9. Olszewski WL. Pathophysiological and clinical observations of obstructive lymphedema of the limbs. In: *Lymphedema* (Clodius L). Georg Thieme (1977).

10. Rutkowski JM, Moya M, Johannes J, Goldman J, Swartz MA. Secondary lymphedema in the mouse tail: Lymphatic hyperplasia, VEGF-C upregulation, and the protective role of MMP-9. *Microvasc. Res.* **72**, 161-171 (2006).

11. Azaripour A, Lagerweij T, Scharfbillig C, Jadczak AE, Willershausen B, Van Noorden CJ. A survey of clearing techniques for 3D imaging of tissues with special reference to connective tissue. *Prog Histochem Cytochem* **51**, 9-23 (2016).

12. Karadjian G*, et al.* Migratory phase of *Litomosoides sigmodontis* filarial infective larvae is associated with pathology and transient increase of S100A9 expressing neutrophils in the lung. *PLoS Negl. Trop. Dis.* **11**, DOI: 10.1371/journal.pntd.0005596 (2017).

13. Wenk P. [the invasion route of the metacyclical larvae of *Litomosoides carinii* chandler 1931 (filariidae)]. *Z. Parasitenkd.* **28**, 240-263 (1967).

14. Bain O, Wanji S, Vuong PN, Marechal P, Le Goff L, Petit G. Larval biology of six filariae of the sub-family onchocercinae in a vertebrate host. *Parasite* **1**, 241-254 (1994).

15. Marechal P, Le Goff L, Petit G, Diagne M, Taylor DW, Bain O. The fate of the filaria *Litomosoides sigmodontis* in susceptible and naturally resistant mice. *Parasite* **3**, 25-31 (1996).

16. Wiszniewsky A*, et al.* The central adaptor molecule TRIF influences *L. sigmodontis* worm development. *Parasitol. Res.* **118**, 539-549 (2019).

17. Güç E*, et al.* Local induction of lymphangiogenesis with engineered fibrin-binding VEGF-C promotes wound healing by increasing immune cell trafficking and matrix remodeling. *Biomaterials* **131**, 160-175 (2017).

18. Kilarski WW, Guc E, Teo JC, Oliver SR, Lund AW, Swartz MA. Intravital immunofluorescence for visualizing the microcirculatory and immune microenvironments in the mouse ear dermis. *PLoS One* **8**, DOI: 10.1371/journal.pone.0057135 (2013).

19. Güç E, Fankhauser M, Lund AW, Swartz MA, Kilarski WW. Long-term intravital immunofluorescence imaging of tissue matrix components with epifluorescence and two-photon microscopy. *Journal of visualized experiments : JoVE*, 10.3791/51388, DOI: 10.3791/51388 (2014).

20. Drinker CK, Field ME. The rate of flow and the pressure of lymph. In: *Lymphatics, lymph and tissue fluid*. The Williams & Wilkins Co (1933).

21. Proulx ST, Ma Q, Andina D, Leroux JC, Detmar M. Quantitative measurement of lymphatic function in mice by noninvasive near-infrared imaging of a peripheral vein. *JCI Insight* **2**, DOI: 10.1172/jci.insight.90861 (2017).

22. Yoffey JM, Coutrice FC. Lymph flow, lymph pressure and lymph composition. In: *Lymphatics, lymph and lymphoid tissue*. 2nd. Harvard University Press (1956).

23. Simonsen PE, Fisher PU, Hoerauf A, Weil GJ. The filariases. In: *Manson's tropical infectious diseases (23rd).* (Farrar J, Hotez PJ, Junghanss T, Kang G, KLalloo D, White N). W.B. Saunders (2014).

24. Baluk P*, et al.* Functionally specialized junctions between endothelial cells of lymphatic vessels. *J. Exp. Med.* **204**, 2349-2362 (2007).

25. Privratsky JR, Newman PJ. PECAM-1: Regulator of endothelial junctional integrity. *Cell Tissue Res.* **355**, 607-619 (2014).

26. Leu AJ, Berk DA, Yuan F, Jain RK. Flow velocity in the superficial lymphatic network of the mouse tail. *Am J Physiol* **267**, H1507-1513 (1994).

27. Safer D, Brenes M, Dunipace S, Schad G. Urocanic acid is a major chemoattractant for the skin-penetrating parasitic nematode Strongyloides stercoralis. *Proc. Natl. Acad. Sci. U. S. A.* **104**, 1627-1630 (2007).

28. Petralanda I, Yarzabal L, Piessens WF. Studies on a filarial antigen with collagenase activity. *Mol. Biochem. Parasitol.* **19**, 51-59 (1986).

29. Scott AL. Lymphatic-dwelling filariae. In: *Tropical medicine* (Nutman TB). Imperial College Press (2000).

30. Kazura JW. Lymphatic filarial infections: An introduction to the filariae. In: *The filaria* (Klei TR, Rajan TV). Kluwer Academic Publishers (2002).

31. McKerrow JH, Caffrey C, Kelly B, Loke P, Sajid M. Proteases in parasitic diseases. *Annu. Rev. Pathol.* **1**, 497-536 (2006).

32. Kusaba T, Fujimaki Y, Vincent AL, Aoki Y. In vitro chemotaxis of *Brugia pahangi* infective larvae to the sera and hemolymph of mammals and lower animals. *Parasitol Int* **57**, 179-184 (2008).

33. Comerford I, McColl SR. Mini-review series: Focus on chemokines. *Immunol Cell Biol* **89**, 183-184 (2011).

34. Jones J. Nematode sense organs. In: *The biology of nematodes* (Lee DL). Taylor & Francis (2002).

35. DeVries ME, Kelvin AA, Xu L, Ran L, Robinson J, Kelvin DJ. Defining the origins and evolution of the chemokine/chemokine receptor system. *J Immunol* **176**, 401-415 (2006).

36. Despommier DD. Chemical trails and the parasites that follow them. *Proc. Natl. Acad. Sci. U. S. A.* **104**, 1447-1448 (2007).

37. Small ST, Tisch DJ, Zimmerman PA. Molecular epidemiology, phylogeny and evolution of the filarial nematode *Wuchereria bancrofti*. *Infect Genet Evol* **28**, 33-43 (2014).

38. Blaxter M, Koutsovoulos G. The evolution of parasitism in *Nematoda*. *Parasitology* **142 Suppl 1**, S26-39 (2015).

39. Quist CW, Smant G, Helder J. Evolution of plant parasitism in the phylum *Nematoda*. *Annu Rev Phytopathol* **53**, 289-310 (2015).

40. Danchin EG, Rosso MN. Lateral gene transfers have polished animal genomes: Lessons from nematodes. *Frontiers in cellular and infection microbiology* **2**, 27 (2012).

41. Croll NA. Behavioural analysis of nematode movement. *Adv. Parasitol.* **13**, 71-122 (1975).

42. Croll NA. Behavioural analysis of nematode movement. *Adv. Parasitol.* **13**, 71-122 (1975).

43. Wallace HR. Wave formation by infective Larvae of the plant parasitic nematode *Meloidogyne Javanica*. *Nematologica* **15**, 65-75 (1969).

44. Izquierdo EJ, Beer RD. From head to tail: A neuromechanical model of forward locomotion in *Caenorhabditis elegans*. *Philos. Trans. R. Soc. Lond., B, Biol. Sci.* **373**, (2018).

45. Langley RR, Fidler IJ. The seed and soil hypothesis revisited - the role of tumor-stroma interactions in metastasis to different organs. *Int J Cancer* **128**, 2527-2535 (2011).

46. Bain O*, et al.* New features on the moults and morphogenesis of the human filaria *Loa loa* by using rodent hosts consequences. *Parasite* **5**, 37-46 (1998).

47. Eberhard ML, Orihel TC. Development and larval morphology of *Loa loa* in experimental primate hosts. *J Parasitol* **67**, 556-564 (1981).

48. Jitpakdi A*, et al.* Variations in microfilariae and infective stages of two types of Wuchereria bancrofti from the Thai-Myanmar border. *J. Helminthol.* **73**, 317-321 (1999).

49. Bain O, Babayan S. Behaviour of filariae: Morphological and anatomical signatures of their life style within the arthropod and vertebrate hosts. *Filaria J* **2**, 16 (2003).

50. Wiig H, Swartz MA. Interstitial fluid and lymph formation and transport: Physiological regulation and roles in inflammation and cancer. *Physiol. Rev.* **92**, 1005-1060 (2012).

51. Long NJ, Wong W-T. *The chemistry of molecular imaging.* John Wiley & Sons, Inc (2015).

52. Amino R*, et al.* Quantitative imaging of *Plasmodium* transmission from mosquito to mammal. *Nat. Med.* **12**, 220-224 (2006).

53. Lee DL. *The biology of nematodes.* Taylor & Francis (2002).
