# Supplementary material for: Inherent biomechanical traits enable infective filariae to disseminate through collecting lymphatic vessels
Source: Nat Commun. 2019 Jul 1;10:2895. doi: 10.1038/s41467-019-10675-2 (PMC6603047; doi:10.1038/s41467-019-10675-2)
Supplement: Supplementary file 3 — Reporting Summary [file 41467_2019_10675_MOESM3_ESM.pdf]

nature research

Corresponding author(s): Witold W Kilarski, NCOMMS-17-25472C

Reporting Summary

Nature Research wishes to improve the reproducibility of the work that we publish. This form provides structure for consistency and transparency in reporting. For further information on Nature Research policies, see Authors & Referees and the Editorial Policy Checklist.

Statistical parameters

When statistical analyses are reported, confirm that the following items are present in the relevant location (e.g. figure legend, table legend, main text, or Methods section).

n/a	Confirmed	
	x	The exact sample size (*n*) for each experimental group/condition, given as a discrete number and unit of measurement
	x	An indication of whether measurements were taken from distinct samples or whether the same sample was measured repeatedly
	x	The statistical test(s) used AND whether they are one- or two-sided<br>*Only common tests should be described solely by name; describe more complex techniques in the Methods section.*
x		A description of all covariates tested
	x	A description of any assumptions or corrections, such as tests of normality and adjustment for multiple comparisons
	x	A full description of the statistics including central tendency (e.g. means) or other basic estimates (e.g. regression coefficient) AND variation (e.g. standard deviation) or associated estimates of uncertainty (e.g. confidence intervals)
	x	For null hypothesis testing, the test statistic (e.g. *F*, *t*, *r*) with confidence intervals, effect sizes, degrees of freedom and *P* value noted<br>*Give P values as exact values whenever suitable.*
x		For Bayesian analysis, information on the choice of priors and Markov chain Monte Carlo settings
x		For hierarchical and complex designs, identification of the appropriate level for tests and full reporting of outcomes
	x	Estimates of effect sizes (e.g. Cohen's *d*, Pearson's *r*), indicating how they were calculated
	x	Clearly defined error bars<br>*State explicitly what error bars represent (e.g. SD, SE, CI)*

Our web collection on statistics for biologists may be useful.

Software and code

Policy information about availability of computer code

Data collection	Both, confocal and live imaging were done with Leica LSM 2.8 software or (for Fig. 5 and Supplementary Fig. 5) with Metamorph ver. 7. Data for Supplementary Fig. 1 were collected with TrueQuant software (PerkinElmer) that operated the Visen Fluorescence Molecular Tomography 2500 device (FMT 2500, Flagship Pioneering).
Data analysis	Image stacks were analyzed with Imaris 7.1 (Bitplane AG, Zürich, Switzerland). Statistical analysis was done with Prism 8.1 (GraphPad, La Jolla, CA). Worms tracking was done in ImageJ (Fiji, current version). Figures were assembled in Illustrator CC or Cytosketch (Cytocede, 310). The movies are encoded in VirtualDub2 (42151, virtualdub.org) with the internal X.264/MPEG-4 AVC 8 bit codec using a single-pass lossless compression in 4:3 aspect ratio (except Movie 10 which is encoded in 16:9 aspect ratio).

For manuscripts utilizing custom algorithms or software that are central to the research but not yet described in published literature, software must be made available to editors/reviewers upon request. We strongly encourage code deposition in a community repository (e.g. GitHub). See the Nature Research guidelines for submitting code & software for further information.

April 2018

Data

Policy information about availability of data

All manuscripts must include a data availability statement. This statement should provide the following information, where applicable:

- Accession codes, unique identifiers, or web links for publicly available datasets
- A list of figures that have associated raw data
- A description of any restrictions on data availability

All data generated or analyzed during this study are included in this published article (and its supplementary information files). Source data are provided as Source Data files. All raw data are available upon request.

Field-specific reporting

Please select the best fit for your research. If you are not sure, read the appropriate sections before making your selection.

☒ Life sciences ☐ Behavioural & social sciences ☐ Ecological, evolutionary & environmental sciences

For a reference copy of the document with all sections, see nature.com/authors/policies/ReportingSummary-flat.pdf

Life sciences study design

All studies must disclose on these points even when the disclosure is negative.

Sample size

We were interested in proving or disproving the all or none effect, therefore the smallest group size for such effect is 3 mice in control and treatment groups (the random probability for 3-0, 0-3 matrix calculated with one-sided Fisher exact test is p=0.05). Nevertheless, the primary lymphedema experiments did not confirm a complete filaria dependence on the lymphatic route. Here, in two experiments, we used all nine (9) available mice with persistent phenotypic edema in the hind limbs age-matched matched with 9 C3H controls.

In the secondary lymphedema group, there were three groups in two experiments: 3 mice in Control groups, 4 sham-operated and 4 (and 6 in the second experiment) in the lymphedema group. In the first experiment, one mouse was lost from the control group before the end of the experiment. In the lymphedema group, four (4) mice that underwent lymphatic occluding surgery but had no change in tail diameter were considered to have functional collecting lymphatics (four mice out of 8 mice in two experiments), therefore these mice were not used in the experiment. Additionally, two out of 6 mice in the second experiment developed tail necrosis and were euthanized. In two experiments, we did not recover any filariae from four mice with palpable lymphedema that were used for the experiment. Countrary, filariae were present in the pleural cavity all of our controls in Fig. 1a (4 unoperated controls and 8 sham-operated mice), but also in control mice in Fig. 1b

Due to lack of prior expectations that would warrant a test of the preliminary hypothesis of filariae and soil-derived nematodes behavior in the skin, the imaging of filariae and soil-nematodes migration in the skin was performed as an observational study, therefore the size of experimental groups could not be pre-determined.

The imaging of subcutaneous spread of filariae was intended to verify whether fluorescent covalent labeling of filariae interferes with their in vivo migration behavior. In each experiment, 50 larvae were injected under the back skin of the mouse. Both experiments are shown in Supplementary Fig. 1.

Confocal microscopy imaging was performed on 14 mice injected with filariae, and 5 mice injected with soil-derived nematodes. Intravital imaging attempts were performed on 11 live-labeled ears injected with filariae, and 9 intact (unstained) ears injected with soil-derived nematodes.

The in vitro experiment that aimed at comparison of the effect of the flow on filariae migration was performed with the same on a single day, in the same maze chamber that was used alternately for static and flow tests with the following order of the tests: static (7 larvae), flow (2 larvae), static (2 larvae), flow (23 larvae), flow (7 larvae). On the other occasion the static experiments was performed once with 6 larvae, and the flow experiment twice with 5 and approximately 45 larvae. The variation of larvae in these tests was the result of variable efficacy of filariae deposition in the Central Station. The tracking of larvae migration for the calculations of RNDs and filariae velocities was performed for runs best matching the number of larvae, that is, static and flow tests that had 7 larvae migrating within the maze of the chamber. The movies of the other tests are available upon request.

The in vitro experiment showing filaria entrapment in the collagen gel was performed twice. The second movie showing entrapment of migratory larvae is available upon request.

Data exclusions

Since the chronic edema is the best indicator of persistent occlusion of lymphatic collectors, only mice with phenotypic lymphedema in the tail (primary) or hind limb (secondary) were used in the experiments and mice that did not show phenotypic edema were excluded were mice that did not show the phenotypic lymphedema. Additionally, mice showing the first sign of tail necrosis were immediately euthanized (two mice in the second experiment).The phenotypic indication of edema: the palpable edema in the tail or tail limb that can be described as: 'doubling of the tail diameter' and 'the diameter of the leg was at least twice the diameter of age-matched wild-type mice for consecutive four weeks.'

In the maze chamber migration experiments dying or dead larvae (larvae, which moves frequency significantly differ from the rest of the group) were not included in the analysis.

For the observational imaging studies, only filariae or soil-derived nematodes that left the inoculation site were used in the analysis.

Replication

The primary and secondary lymphedema experiments were repeated once (all data are pooled on the graphs in Fig. 1 a-b).

Primary lymphedema (Chy3/C3H mice). In two experiments we used all the Chy3-C3H mice that showed persistent lymphedema in the hind leg (mice were collected during 6 month breeding period). Hence, the first experiment had 5 (the 6th mouse died during the anesthesia before the injection of filariae) and the second experiment was carried with 4 mice with phenotypic lymphedema. The number of C3H control mice in each experiment was matched to the mouse number in the corresponding lymphedema group. The C3H mice were selected from

aphenotypic littermates of mice with persistent hind-limb edema.
Secondary lymphedema (surgery, C57BL/6) mice. Two experiments with the following groups: 3 mice in Control groups (in the first experiment one mouse died from unknown causes before the end of the experiment), 4 sham-operated and 4 (and 6 in the second experiment) 'lymphedema group.' In the lymphedema group, four (4) mice that underwent lymphatic occluding surgery but had no change in tail diameter were considered to have functional collecting lymphatics (four mice out of 8 mice in two experiments), therefore these mice were not used in the experiment.

The whole body near-infra-red imaging was performed twice, each with 50 subcutaneously injected labeled filariae.

Confocal microscopy imaging was performed on 14 mice injected with filariae, and 5 mice injected with soil-derived nematodes. Intravital imaging attempts were performed on 11 live-labeled ears injected with filariae, and 9 intact (unstained) ears injected with soil-derived nematodes.

The in vitro experiment that aimed at comparison of the effect of the flow on filariae migration was performed with the same on a single day, in the same maze chamber that was used alternately for static and flow tests with the following order of the tests: static (7 larvae), flow (2 larvae), static (2 larvae), flow (23 larvae), flow (7 larvae).
The in vitro experiment showing filaria entrapment in the collagen gel was performed twice. The second movie showing entrapment of migratory larvae is available upon request.
The source data files are enclosed with this submission.

Randomization

Since the phenotypic edema (in the footpad, Fig. 1a) or tail (in the tail, Fig. 1b) is the best indicator of persistent lymphatic occlusion (visible to the naked eye), subject randomization before injection of filariae in the tail of footpad could not be performed.
As there were no experimental groups, no randomization could be done for the observational study.

Blinding

For lymphedema experiments, two technicians who were not involved in the study euthanized the animals, performed pleural washes and counted filariae under the stereomicroscope.
As observational studies, the imaging experiments could not be blinded. However, due to the lack of pre-existing hypothesis in these studies, the subjective effect of the experimenter (all procedures from filaria isolation to image analysis were done by WWK) was minimized.
In vitro maze chamber migration experiment was performed by MP and analyzed by MP, MAS, and WWK.

Reporting for specific materials, systems and methods

Materials & experimental systems

n/a	Involved in the study
☒	☐ Unique biological materials
☐	☒ Antibodies
☒	☐ Eukaryotic cell lines
☒	☐ Palaeontology
☐	☒ Animals and other organisms
☒	☐ Human research participants

Methods

n/a	Involved in the study
☒	☐ ChIP-seq
☒	☐ Flow cytometry
☒	☐ MRI-based neuroimaging

Antibodies

Antibodies used

We don't have lot numbers of antibody used. These commercial antibodies are used on regular bases, and frequently new antibodies were bought over the years. In our experience, these structural antibodies work independently on the lot as described by the manufacturer, so any deviation from the routine would have been immediately noticed.
Biotinylated polyclonal anti-collagen IV antibody (Abcam; ab6581); monoclonal rat anti-VE-cadherin (BD, 550548, clone 7B4), hamster anti-mouse PECAM-1 (Genetex, clone 2H8, GTX74943), rabbit anti mouse Lyve-1 (Reliatech, polyclonal, 103-PA50), streptavidin Alexa 647 (Invitrogen, A21110), polyclonal secondary antibody from Invitrogen): donkey anti-rat Alexa 594 (Invitrogen , A21209), goat anti-hamster Alexa 488 (Invitrogen , A21110), donkey anti-rabbit Alexa 488 (Invitrogen , A21206).

Validation

No specific validation was performed on any commercially-available antibodies from the above suppliers. The structural stainings are characteristic, so any divergence from the routine in regular experiments would be immediately noticed.

Animals and other organisms

Policy information about studies involving animals; ARRIVE guidelines recommended for reporting animal research

Laboratory animals

C3H wild-type and Chy3/C3H heterozygous mice (chromosomal deletion that includes VEGF-C) were purchased from the Jackson Laboratory. Tail lymphedema was induced in C57BL/6 mice (the Jackson Laboratory). All imaging experiments with filariae and nematodes were done using BALB/c (the Jackson Laboratory or Charles River) females aged between 12 and 20 weeks. A comparison between lymphatic endothelial cells Tomato (red) reporter protein and lymphatic basement membrane staining was made with a Prox1-Tomato mouse (Charles River).

Wild animals	L. sigmodontis was isolated from mites that were sent from Museum national d'Histoire naturelle, Paris, France.
Field-collected samples	Free-living nematodes were collected from soil samples outside the Eckhard Research Center building, University of Chicago. As we stated in the Method section: 'Free-living nematodes were isolated from soil samples collected at the University of Chicago, the location not visited by the wild, household or farm animals. Actively moving nematodes were purified from the soil samples using the Baermann funnel method. Briefly, large particles were first removed from 100 g soil samples by mixing soil-samples with 300 ml of deionized water and passing the slurry through a funnel-shaped No. 20 sieve (850 µm mesh size). The same mesh sieve acted as a screen after it was placed within a larger funnel fitted with 5 cm of rubber tubing, which was sealed with a hemostat. A Kleenex tissue paper was placed on the screen, and deionized water was poured into the funnel to cover approximately half of the fitted screen. Soil samples were then poured onto the screen, and nematodes were collected in the first 50-ml fraction from the rubber tubing 48 hours later. Nematodes were spun down at 500 g and hand-picked with a 200 µl pipette from the residual 5 ml of water. Collected nematodes were kept in deionized water at 4°C for up to 24 hours. Prior to the staining with TRITC, the nematodes were washed twice with PBS, pH 7.4, and transferred to the reaction vial with 100 µl PBS.'.
