# Supplementary material for: Inherent biomechanical traits enable infective filariae to disseminate through collecting lymphatic vessels
Source: Nat Commun. 2019 Jul 1;10:2895. doi: 10.1038/s41467-019-10675-2 (PMC6603047; doi:10.1038/s41467-019-10675-2)
Supplement: Supplementary file 4 — Description of Additional Supplementary Files [file 41467_2019_10675_MOESM4_ESM.pdf]

Supplementary movies

The movies were encoded in VirtualDub2 with the internal X.264/MPEG-4 AVC 8 bit codec using a single-pass lossless compression in 4:3 aspect ratio (except Movie 10 which is encoded in 16:9 aspect ratio).

Supplementary movie 1

Labeled filariae escape the subdermal inoculation site

A whole-body imaging sequence shows the subcutaneous spread of filariae injected in the lumbar region of the mouse (analyzed in Supplementary Figure 1b). The first 65 images were taken every 3 to 5 min immediately after the inoculation, and the last 4 images were taken 20 h after the inoculation. The image sequence was registered using the RigidBody option of Fiji (ImageJ) StackReg plugin (Biomedical Imaging Group, EPFL). The gradient lookup table represents a single imaging near-infra-red channel with blue color showing an autofluorescent outline of a mouse and a red, the strong signal from filaria and liver autofluorescence. The movie is played at 3 fps.

Supplementary movie 2

Only a fraction of filaria larvae leave the intradermal injection site

Intravital imaging of the intact ear 2.5 h after filariae inoculation. Most skin-injected larvae remained at the inoculation pocket. The 4.3 min movie, encoded from images taken every 30 s, is played at 30 fps.

Supplementary movie 3

Filariae require solid support to migrate within the matrix

In vitro imaging of (unlabeled) filariae moving across the artificial collagen matrix. Two infective larvae entrapped in 3 mg/ml rat tail collagen type I gel covered with cell culture media. The larva in the lower part of the video moved during gel polymerization which leads to the formation of a fluid-filled matrix void. Larva at the top of the movie was temporarily paralyzed with 1 mg/ml of lignocaine before being embedded in the collagen sol. The first larva that moved within a fluid-filled gel pocket could not spring back from the surrounding collagen to initiate its move across the gel. In contrast, the second larva, which movements were paralyzed during gel polymerization, became embedded in the gel. After the effect of the lignocaine wore off, the larva could migrate within the gel, first by springing back from the surrounding matrix, then by the drilling movement of its entire body. imaging. The 5-min movie shows bright-field (phase-contrast) imaging with images taken every 0.2 s. The movie is played at 250 fps.

Supplementary movie 4

Overview of the intravital imaging window and filaria inoculation site

Intravital imaging of the surgically exposed and stained dorsal dermis of the ear. Vessels of the dorsal ear skin are stained within the window created by excision of ventral skin and the underlying cartilage. Filariae are injected in the dorsal skin at the distal fragment of the ear. Only a few larvae managed to escape from the injection site (inoculation pocket) and entered the stained area. Even then, some of these larvae left the skin after migrating towards the surface of the stained dermis (i.e., a fast-moving larva that slides over the surface of the stained skin) Larva that left the skin could not re-enter it. This movie is combined from three shorter videos (respectively, 1, 3, and 15 min long) taken at different magnification and at different time intervals: images were taken every 4 s (the first, one-min-long fragment of the movie), every 6 s (the second, 3-min-long movie fragment), and 13 s (the third, 15-min-long movie fragment). Red-filaria, green-collagen IV. The movie is played at 10 fps.

Supplementary movie 5

Filariae cannot enter the exposed dermis from the fluid media

Intravital imaging of the exposed and stained dorsal ear dermis. FITC-labeled filariae overlaid on the surface of the exposed and live-stained for collagen IV dorsal dermis could only slide over the surface but could not enter the dermis. Red, filaria; green, collagen IV. At the end of the movie the same sequence showing only red filaria channel is re-played. The 8.8 min movie, with images taken every 10 s, is played at 10 fps.

Supplementary movie 6

Migratory filaria larva stranded in the dermis

Intravital imaging of the exposed and stained dorsal ear dermis. The larva that entered the stained skin area was stranded in the tissue between vessels for 2 h. The red glow is a result of the rapid increase in autofluorescence during the accidental drain of the media covering the ear. Red, filaria; green, live-stained basement membrane (collagen IV). The 62-min movie, with images taken every 120 s, is played at 5 fps.

Supplementary movie 7

Intermittent pattern of filariae migration in the dermis

Intravital imaging of the exposed and stained dorsal ear dermis. Filariae were inoculated in the distal, unstained region of the ear that is covered with a remaining fragment of ventral skin. A single larva entered the exposed and stained area of the dorsal dermis and advanced at variable speed and directionality. The larva spent most of its time in the dermis on backward-forward movements interleaved with rare episodes of migratory advances. Red, filaria; green, live-stained BM (collagen IV). The 5.5 h movie, with images taken every 60 s, is played at 20 fps.

Supplementary movie 8

Filaria migrating in the dermis enters a lymphatic collecting vessel

Intravital imaging of the exposed and stained dorsal ear dermis. Filariae were inoculated in the distal, unstained region of the ear that is covered with a remaining fragment of ventral skin. Filaria left the unstained inoculation area 40 min after inoculation. The larva first unsuccessfully burrowed into the blood capillary and then pushed forward towards the lymphatic pre-collector. After crossing the collector 3 min later, the larva rapidly entered the vessel. Before inoculation, the surgically exposed window of the dorsal dermis was stained for collagen IV (BM, green); red, filaria. At the end of the movie, the video is re-played with lymphatic entry location zoomed-in. The out-of-focus dead larva (top-left corner) migrated to its location before the imaging started. The 1.5-h movie, with images taken every 1 min, is played at 20 fps.

Supplementary movie 9

Filaria migrates across the ear within a lymphatic collecting vessel

Intravital imaging of the exposed and stained dorsal ear dermis. Filariae were inoculated in the distal, unstained region of the ear that is covered with a remaining fragment of ventral skin. Within this hidden region, a single inoculated larva migrated out of the inoculation pocket into the dermis and enter the lymphatic pre-collector. The larva appeared migrating within lymphatics 6 min after the inoculation. The dorsal skin was live-stained for collagen IV (BM, green; note the contraction/relaxation of the stained ear skeletal muscles (center). The movie recording started 3 min after inoculation of the larvae. Red, filaria; green, collagen IV. The 23-min movie, with images taken every 20 s, is played at 5 fps.

Supplementary movie 10

Deformation of the lymphatic wall during the passage of larva

Intravital imaging of the exposed and stained dorsal ear dermis. The same image sequence as in Supplementary movie 9 showing only the collagen IV fluorescence channel. The movie depicts the mechanical deformation of lymphatic basement membrane during the passage of the filaria. Green, collagen IV, red, filaria. The 23-min movie, with images taken every 20 s is played at 5 fps.

Supplementary movie 11

Fluid flow directs filariae migration

In vitro effect of fluid currents on the directionality of filariae migration. In the presence of the fluid currents (current from the Nord-East to South-West), filariae displayed a positive current tropism migrating towards the S-W exit. Filaria larvae were injected into the Central Station of the microfluidic maze device. A single, possibly dying (slow advance and weak bending movements) larva is migrating forward and not responding to the

flowing fluid. Bright field (phase-contrast) imaging. The 28-min movie, with images taken every 10 ms, is played at 104 fps.

Supplementary movie 12

Filariae move randomly in the absence of fluid currents

In vitro effect of fluid currents on the directionality of filariae migration. In the absence of fluid currents, filariae migrated randomly within channels of the microfluidic device. Filariae were injected into the Central Station of the microfluidic maze device. Bright field (phase-contrast) imaging. The 18-min movie, with images taken every 10 ms, is played at 104 fps.

Supplementary movie 13

Variable staining of soil-derived nematodes

In vitro movement of variably TRITC-labeled soil-derived (free-living and phytoparasitic) nematodes. Red, TRITC-labeled nematodes (highlights) and tissue autofluorescence (midtones). The 8-s movie, with images collected every 0.1 s, is played at 5 fps.

Supplementary movie 14

Only a fraction of nematodes left the inoculation pocket

Through-the-skin intravital imaging in the intact dorsal ear inoculated with TRITC-labelled soil-derived nematodes. The imaging shows the inoculation site of soil-derived nematodes 30 min after intradermal injection. Similar to filaria larvae, most skin-injected soil-derived nematodes remained within post-injection skin pocket. Nematodes that left the skin injection pocket were at the permanent move, but their migratory progress was limited to rare bursts interleaved with long periods of negligible migratory activity. Red, nematodes; green, tissue autofluorescence. The 17-min movie, with images taken every 15 s, is played at 10 fps.

Supplementary movie 15

Transient effect of phototoxicity on the movement of nematodes *in vivo*

Through-the-skin intravital imaging in the intact dorsal ear inoculated with TRITC-labelled soil-derived nematodes. Two joined videos (first 25 s, second 48 s) showing a single nematode exposed continuously to the excitation light. In contrast to filaria imaging, toxic free-radicals could not be scavenged with the application of isotonic sodium ascorbate. Instead, the cease of the movement activity of the imaged nematode (16-25 s in the movie) triggered a 5-10 min imaging pause allowing the nematode to recover. The imaging that resumed 5 min later with reduced power of the excitation light shows the continuous movement of the same nematode that lasted for 48 s. Red, TRITC-labeled nematodes (highlights) and tissue autofluorescence (midtones). Frames were collected every 0.25 s, and the movies are played at 10 fps.

Supplementary movies 16

Soil-derived nematode migrates through the dermis (first example)

Through-the-skin intravital imaging in the intact dorsal ear inoculated with TRITC-labelled soil-derived nematodes. Multidirectional advances of soil-derived nematode migrating in the skin were interleaved with periods of negligible migratory progress. Despite the constant movement of the nematode migrating through the skin, its net advances were made at rare and sudden migratory bursts. Red, TRITC-labeled nematodes (highlights) and tissue autofluorescence (midtones and shadows). The 38-min movie with images taken every 15 s played at 10 fps.

Supplementary movies 17

Soil-derived nematode migrates through the dermis (second example)

Through-the-skin intravital imaging in the intact dorsal ear inoculated with TRITC-labelled soil-derived nematodes. Multidirectional advances of soil-derived nematode migrating in the skin were interleaved with periods of negligible migratory progress. Despite the constant movement of the nematode migrating through the skin, its net advances were made at rare and sudden migratory bursts. Red, TRITC-labeled nematodes

(highlights) and tissue autofluorescence (midtones and shadows). The 31-min movie, with images taken every 7 s played at 10 fps.

Supplementary movie 18

Rapid and unidirectional migration of soil-derived nematode through the ear dermis

Through-the-skin intravital imaging in the intact dorsal ear inoculated with TRITC-labelled soil-derived nematodes. Sudden and rapid unidirectional migratory bursts of the nematode were interleaved with comparable in time, migratory breaks. The nematode that migrates the longest path begins its migration in the middle of the movie. The continued migration of the nematode is shown after the 7-min imaging pause in the Supplementary movie 19. Red, nematodes; green, autofluorescent glow of the skin, black, blood-filled non-autofluorescent blood vessels. Time-lapse images were taken for 19 min every 15 s, and movies is played at 5 fps.

Supplementary movie 19

Continued migration of the nematode from Supplementary movie 18 that started after 7 min pause. Time-lapse images were taken for 11-min every 15 s, and movie is played at 5 fps.

Supplementary movie 20

Soil-derived nematode located in the subcapsular sinus of the lymph node

Ex vivo imaging of the ear draining superficial cervical lymph node excised 6 h after the soil-derived nematodes were injected in the mouse ear. The arrow points to the actively moving nematode under the capsule of the lymph node. At the end of the movie, the zoomed-in location of the nematode is played again. Red, nematode over the red autofluorescent glow of the lymph node. Frames were collected every 0.25 s, and the movies are played at 10 fps.
